# Membrane depolarization and aberrant lipid distributions in the neonatal rat brain following hypoxic-ischaemic insult

**DOI:** 10.1038/s41598-018-25088-2

**Published:** 2018-05-03

**Authors:** Dominika Luptakova, Ladislav Baciak, Tomas Pluhacek, Anton Skriba, Blanka Sediva, Vladimir Havlicek, Ivo Juranek

**Affiliations:** 1 Institute of Microbiology of the Czech Academy of Sciences, Prague, 142 20 Czech Republic; 2 Institute of Experimental Pharmacology and Toxicology, CEM of the SAS, Bratislava, 841 04 Slovakia; 3 Slovak University of Technology, Central Laboratories, Bratislava, 812 37 Slovakia; 4 Regional Centre of Advanced Technologies and Materials, Department of Analytical Chemistry, Olomouc, 771 47 Czech Republic

## Abstract

Neonatal hypoxic-ischaemic (HI) encephalopathy is among the most serious complications in neonatology. In the present study, we studied the immediate (0 hour), subacute (36 hours) and late (144 hours) responses of the neonatal brain to experimental HI insult in laboratory rats. At the striatal level, the mass spectrometry imaging revealed an aberrant plasma membrane distribution of Na^+^/K^+^ ions in the oedema-affected areas. The failure of the Na^+^/K^+^ gradients was also apparent in the magnetic resonance imaging measurements, demonstrating intracellular water accumulation during the acute phase of the HI insult. During the subacute phase, compared with the control brains, an incipient accumulation of an array of *N*-acylphosphatidylethanolamine (NAPE) molecules was detected in the HI-affected brains, and both the cytotoxic and vasogenic types of oedema were detected. In the severely affected brain areas, abnormal distributions of the monosialogangliosides GM2 and GM3 were observed in two-thirds of the animals exposed to the insult. During the late stage, a partial restoration of the brain tissue was observed in most rats in both the *in vivo* and *ex vivo* studies. These specific molecular changes may be further utilized in neonatology practice in proposing and testing novel therapeutic strategies for the treatment of neonatal HI encephalopathy.

## Introduction

Neonatal hypoxic-ischaemic encephalopathy (HIE) is among the most common neurological complications in neonatology. Due to its high mortality and chronic morbidity in surviving infants, HIE represents a serious socioeconomic issue^1^. The incidence of HIE is 1–4 per 1000 live births in developed countries and is usually higher in developing countries^2^. In approximately 25% of survivors, hypoxic-ischaemic (HI) brain injury can result in a variety of severe neuro-psychological symptoms, such as cerebral palsy, seizures, epilepsy, mental retardation, learning and/or behavioural disorders, etc^3^. The early phase of HI brain insult is characterized by primary energy metabolism failure caused by an impaired cerebral blood flow and insufficient oxygen supply^4^. After the re-oxygenation of the brain and the ostensible recovery of its functions, secondary energy failure may occur. The secondary injury is considered responsible for the long-lasting consequences of HI insult^5^. At the beginning of HI insult, the brain metabolism switches to the anaerobic mode due to the low oxygen availability. The inhibition of oxidative phosphorylation leads to a decrease in the phosphocreatine and ATP levels and an increase in the inorganic phosphate and lactic acid levels. The hypoxic inhibition of ATP-dependent ion pumps leads to cell membrane depolarization, which is mostly due to potassium ion leakage from the cell and electrochemically favoured Na^+^ and Ca^2+^ influx into the cell. This depolarization, in turn, leads to water entry and accumulation inside the cell and, thus, cell swelling. The cell swelling along with the cytosolic calcium overload may ultimately result in cell lysis^6,7^. Hence, the water accumulation inside the cell underlies the evolution of cytotoxic oedema, which is an early sign of HI insult^8^.

During the reperfusion/re-oxygenation phase, the brain metabolism may appear to recover. The hypoxia-induced cytosolic calcium overload may diminish due to increased calcium sequestration inside intracellular organelles, such as the endoplasmic reticulum and mitochondria. However, this sequestration may lead to calcium overload in these organelles, resulting in their dysfunction. Moreover, the reintroduction of oxygen into posthypoxic tissue can result in the overproduction of reactive oxygen and nitrogen species that may exacerbate the primary HI injury^9^. Due to the mitochondrial calcium overload, the mitochondria may functionally and structurally disintegrate, resulting in secondary energy metabolism failure^4,7,10^, and the activation of calcium-dependent phospholipases, proteases and endonucleases and the resulting lipid peroxidation, protein oxidation, and fragmentation of DNA/RNA are responsible for triggering cell death processes^6,10,11^. The overall process is characterized by excitotoxicity, progressively evolving brain oedema and exacerbation of the primary insult. The HI-induced break-up of cellular membranes causes the release of membrane-derived lipid metabolites^12^.

For a timely diagnosis of neonatal brain HI insult and the application of effective therapeutic interventions, the period between the primary and secondary energy failures is crucial. This period is generally referred to as “the window of opportunity”^13^. In neonatology practice, newborns who meet the inclusion criteria (i.e., gestation age ≥ 36 weeks, Apgar score < 5 at 10 minutes, severe acidosis (pH < 7), umbilical cord base deficit ≥16 mmol/L and signs of foetal distress/acute perinatal events) should be considered for mild hypothermia (32–34.5 °C) therapy, which is the only currently approved therapy. However, therapeutic hypothermia is not very effective^14–16^. Only one in every 8–9 HI-affected newborns treated with therapeutic hypothermia survives without moderate or severe consequences, and up to half of all hypothermia-treated infants may suffer permanent neurological consequences^17^.

In the present study, we focused on cellular and molecular processes occurring in acute, subacute and late stages of HI brain insult in newborn rats. While searching for potential predictive biomarker(s) of the neonatal brain HI injury, we particularly focused on brain lipidome by means of multimodal *in vivo* magnetic resonance imaging (MRI), *ex vivo* light microscopy and mass spectrometry imaging (MSI) techniques. MSI combined matrix-assisted laser desorption/ionization (MALDI) and laser ablation inductively coupled plasma mass spectrometry (LA-ICP-MS). We report a close association between the early evolution of oedema and an aberrant Na^+^ and K^+^ distribution, reflecting cell membrane depolarization. Importantly, we identified distinct lipid metabolites that could be used prospectively as biomarkers reflecting the severity of neonatal HIE.

## Results and Discussion

### *In vivo* monitoring of the acute, subacute and late stages of HIE

**Figure 1:**
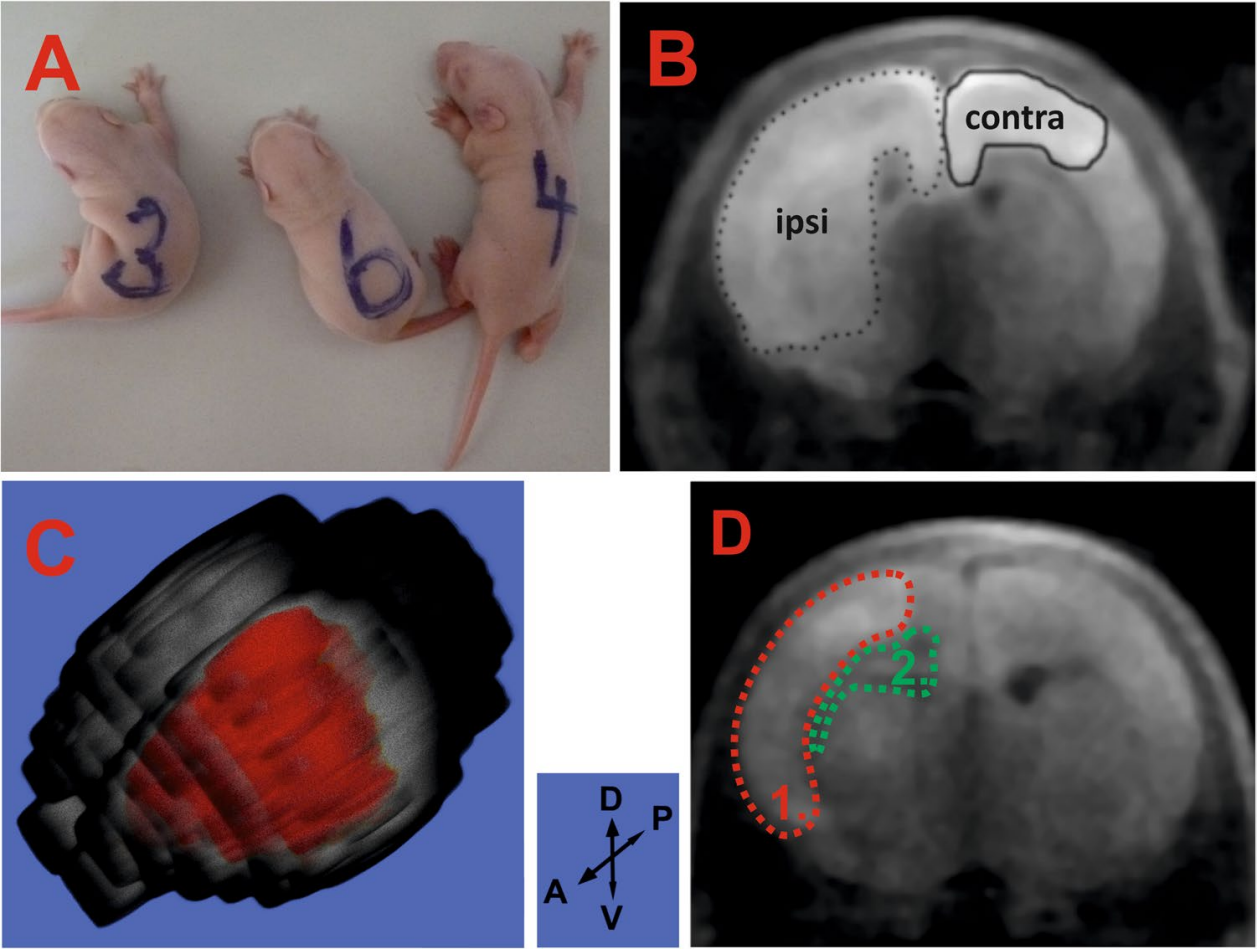
Effects during the acute and subacute stages of HI insult. **(A)** – insulted pups curved to the left (#3 and 4), while the sham pups did not (#6). **(B)** – cytotoxic oedema development in the ipsilateral hemisphere (dotted line) and contralateral hemisphere (bold line) was detected immediately after HI insult at the striatal level (pup #16). **(C)** – 3D image of the brain depicting the extent of the lesion (in red) in the ipsilateral hemisphere (pup #7); D-V and A-P black arrows indicate the dorso-ventral and anterior-posterior directions, respectively. **(D)** – subacute stage: areas of simultaneously occurring vasogenic and cytotoxic oedema are shown in green and red, respectively (pup #9).

After HI insult, the HI-affected rats curved to the left side from the central body axis as an immediate response to the insult, while the non-affected sham control rats remained in an apparently normal position (Fig. 1A). The development of cerebral oedema was monitored *in vivo* by diffusion-weighted (DW) magnetic resonance imaging (DWI). Cytotoxic oedema affected the cortex, corpus callosum, external capsule, striatum, hippocampus, hypothalamus, thalamus, lateral septal nuclei and amygdala (Supplementary Figure S1D). The average size of the cytotoxic oedema-affected area in the ipsilateral hemisphere (seven biological replicates) was 11.9 ± 1.2% (mean ± standard error of the mean (SEM)) (Supplementary Table S1). Intriguingly, in two HI-insulted rats, the cytotoxic oedema spread to the contralateral hemisphere, mainly affecting the cortex, and represented 1.7 ± 1.2% of the total hemisphere size (Supplementary Figure S1B). Cytotoxic oedema lesions with the typical massive intracellular water accumulation provided bright hyperintensive signals on DWI (Fig. 1B). The 3D reconstruction was built from consecutive slices (Fig. 1C). No oedema development was observed in the control animals (n = 6).

At 36 hours after HI insult, the cytotoxic oedema size significantly decreased, and cytotoxic oedema-related hyperintensities were observed only in the cortex of the ipsilateral hemisphere. However, the hypointensities characteristic of the vasogenic type of oedema began to appear in the lateral ventricle, hippocampus and external capsule. At this time point, both the cytotoxic and vasogenic types of oedemas were simultaneously present, which is referred to as biphasic oedema (Fig. 1D**)**. In the sham animals, no pathological changes were observed on DWI, and both hemispheres had an identical appearance.

During the late stage of the HI insult (144 hours), only two of the seven surviving rats developed progression of the brain lesion as documented by the larger extent of the vasogenic oedema, which was indicative of a negative outcome for these animals. An increased hypointensive signal was observed in the lateral ventricle, hippocampus, striatum, corpus callosum, external capsule and cortex brain regions. No characteristic changes were observed in the other five rats, and their initial brain lesions decreased to the zero level (Supplementary Figure S1C).

### Cell membrane depolarization as an immediate response to HI insult monitored *ex vivo*

**Figure 2:**
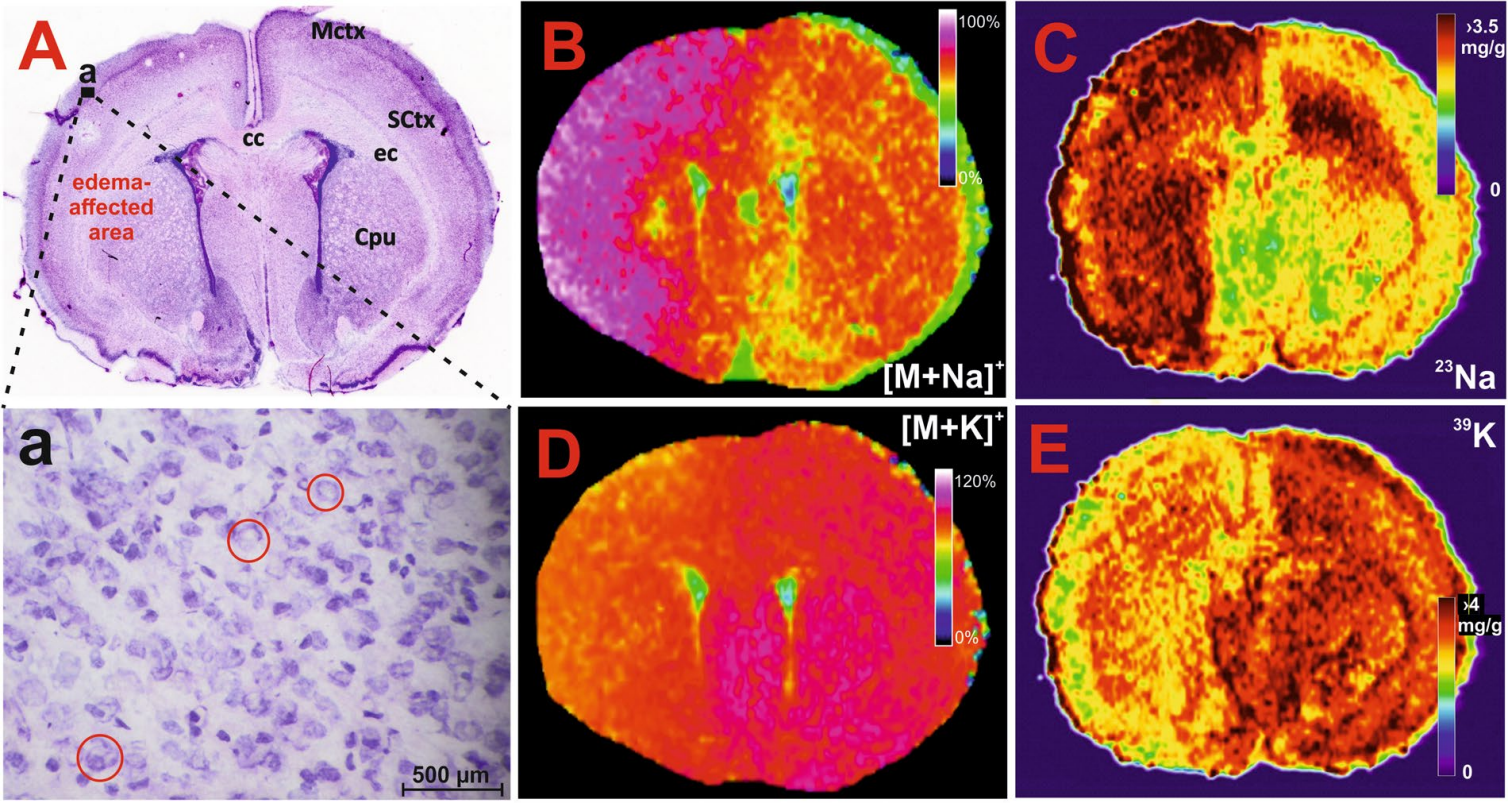
Histological and MSI assessment of the HI-insulted neonatal rat brains during the acute stage of HIE. Panel A: Haematoxylin-eosin stained section demonstrating the early cellular response; a – expanded region of the ipsilateral hemisphere (indicated by the black rectangle shown in (A)) depicting the cytotoxic oedema-afflicted neurons with signs of swelling and vacuolization (red circles). MALDI MS images showing the altered distribution of PC (16:0/16:0) in the sodiated (panel B, *m/z* 756.550) and potassiated (panel D, *m/z* 772.525; signal was overscaled to 120% to provide better contrast) form. Quantitative LA-ICP-MS images of the elemental sodium (panel C) and potassium (panel E) distribution (for details see Supplementary Figure S4). MCtx (motor cortex), cc (corpus callosum), SCtx (somatosensory cortex), ec (external capsule), Cpu (caudate putamen = striatum). Different image scaling (B/D versus C/E) are due to different FlexImaging (Bruker Daltonics, Bremen, Germany) and ImageLab^42^ programs.

The *ex vivo* changes in neuronal vacuolization were detected by microscopy as numerous shapeless and optically empty vacuoles in the cytoplasm and were observed primarily in the cortex during the early stage of HI insult (Fig. 2A). Cytotoxic oedema in the neuronal cells manifested as a triangular nuclei shape, nuclear membrane thickening and neuronal swelling (Fig. 2Aa). No traces of neuronal cell death were observed. In all HI brain samples, signs of oedema were detected in the cortex, striatum, external capsule and corpus callosum of the ipsilateral hemisphere. In four HI samples, oedema spread to the contralateral hemisphere, primarily affecting the cingulate cortex, motor cortex and corpus callosum. Compared to the control animals, a visible compression of the ventricular system was typically observed in all HI brains. Notably, the non-affected areas possessed features that were similar to those in the control sham brains.

Phosphatidylcholines (PCs) dominated the positive ion mode MALDI spectra of both the HI-insulted (Supplementary Figure S2A) and sham brains. During the early response of the brain to the HI insult, the intensities of the PCs at *m/z* 728.520, 754.536, 756.551, 782.567, and 784.583 increased, while the intensities at *m/z* 744.494, 770.510, 772.525, 798.541 and 800.557 decreased (Supplementary Table S2). Individual species were defined by performing a CycloBranch exact mass search^18^ against the LipidMaps database. Compared with the control brains, the sodium adducts of the PCs were increased, while the potassium adducts were decreased in the HI-affected brains. The distribution of PC (16:0/16:0) is depicted in Fig. 2B and D. The structure of the lipids was acquired by collision-induced dissociation MS analysis (see Supplementary Figure S3 as an example).

The molecular profiles of the lipids cationized with Na^+^ or K^+^ matched well with the elemental profiles of sodium and potassium. According to the quantitative LA-ICP-MSI analysis, the sodium concentration was approximately 1.5-fold higher at the striatal level of the HI-affected brain area (Fig. 2C) and the potassium concentration was significantly lower (Fig. 2E) than the uniform, symmetric distribution of these elements in the control brain. In the LA-ICP-MSI analysis, a sufficient linear range was achieved for both the sodium and potassium calibration plots using dried droplet calibration standards (Supplementary Figure S4).

The most abundant signals in the negative ion MALDI mass spectra were attributed to phosphoethanolamines (PE), phosphatidic acids (PA), phosphoinositoles (PI) and monosialoganglioside GM1(d18:1/18:0) (*m/z* 1544.863) (Supplementary Figure S2B). The most intense peak observed in all spectra occurred at *m/z* 885.549 and was ascribed to PI (38:4). The phosphate-containing molecules ADP (*m/z* 426.021) and AMP (*m/z* 346.053) were detected as [M-H]^−^ ions, and their structures were assigned using both exact mass and tandem mass spectrometry (Supplementary Figures S5 and S6). In the *m/z* mass range from 970 to 1070, an array of *N*-acylphosphatidylethanolamine (NAPE) molecules was detected in the spectra of the HI-affected brains. During the acute stage following HI insult, the relative abundance of the NAPEs was approximately 2-fold higher in the affected animals than that in the control sham brains (n = 6).

### Subacute changes in the brain lipidome

**Figure 3:**
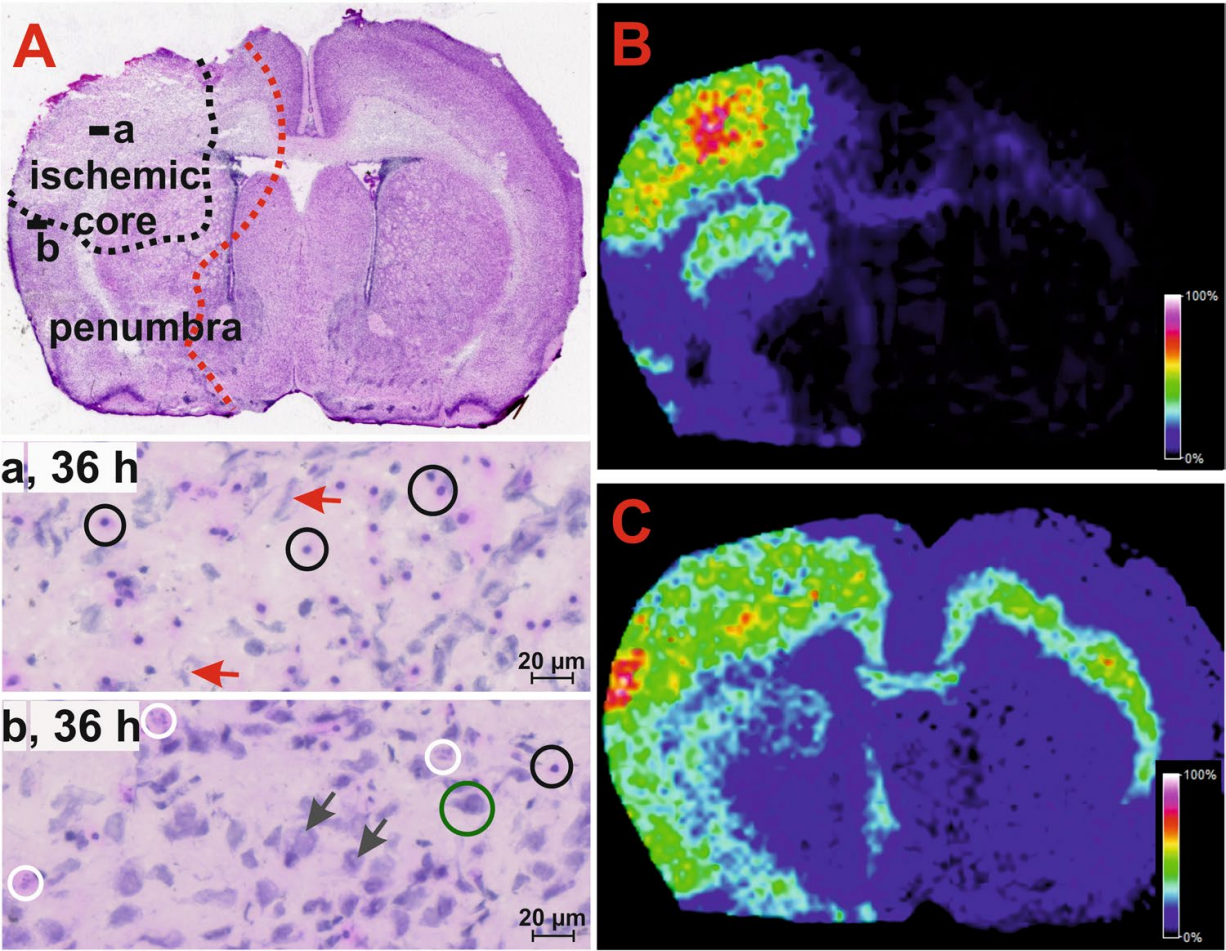
Subacute response to HI insult in the neonatal rat brain. **(A)** Haematoxylin-eosin stained section showing the ischaemic core of the lesion containing large-bodied astrocytes with an eosinophilic cytoplasm (cells indicated by black circles in the both expanded areas) and necrotic neuronal debris (red arrows, panel a); penumbra area (panel b) showing necrotic (white circles) and shapeless (black arrows) cells and intact neurons (green circle). MALDI MS images showing the altered distribution of NAPE (54:4) and GM2 (d18:1/18:0) in the affected area detected as deprotonated molecules at *m/z* 1004.768 (**B**) and 1382.805 (**C**), respectively.

The subacute response was analysed at the 36-hour time interval and involved significant damage to neuronal tissue, with signs of liquefactive necrosis resulting in the occurrence of large-body astrocytes with enlarged vesicular nuclei, an abundant eosinophilic cytoplasm and neuronal debris (Fig. 3A). The myelin in the necrotic area appeared pale due to the surrounding oedema fluid, and many macrophages were present. The necrotic area was nearly identical to the actual ischaemic core of the lesion, which was surrounded by penumbra comprising shapeless and blobbing neurons and/or neurons undergoing necrotic cell death. The severe lesions were predominantly located in the ipsilateral hemisphere of the HI-insulted brain, and the ischaemic core typically affected the motor cortex, somatosensory cortex, external capsule and upper portion of the striatum. In the corpus callosum, the characteristic initial stage of cell degeneration was observed as pale or slightly pink tissue. In the contralateral hemisphere, the corpus callosum and external capsule were the only structures affected.

The results of the positive ion mode MALDI MSI measurements were similar to those observed during the acute stage of HI injury. Aberrant distribution of Na^+^ and K^+^ adducts of the PCs was detected in the severely HI-affected brain areas, where the Na^+^ adducts of the PCs dominated the analogous K^+^ adducts. Thus, the persistent membrane depolarization was associated with the accumulated oedema fluid (Supplementary Figure S7).

**Figure 4:**
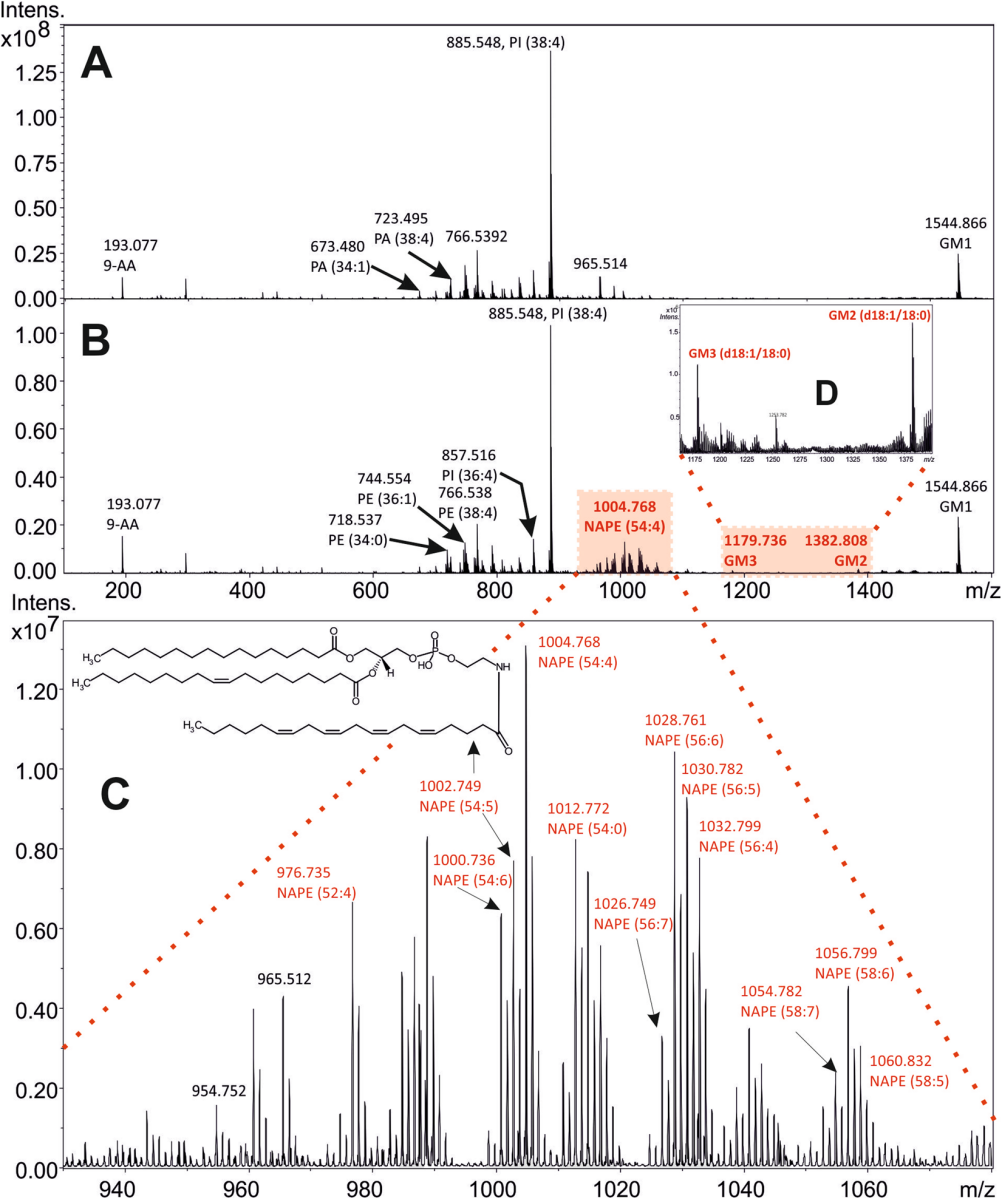
Subacute alterations in the phospholipid profile in the HI-affected area of the neonatal rat brain. Illustrative spectra of the entire mass range acquired by negative ion MALDI from the ischaemic core (panel B) and the corresponding area in the sham control rat brain (panel A). Detailed arrays of NAPE and monosialoganglioside molecules are depicted in panels C and D, respectively.

The negative ion MALDI MSI revealed that the HI-insulted brain significantly differed from the sham brain. Aberrant distribution of NAPEs, GM2, and GM3 (Supplementary Table S3) was found in four of the six HI-insulted brains (Fig. 4B). Importantly, these aberrations corresponded to the severely degenerated tissue of the ischaemic core affecting the motor, somatosensory cortex and upper portion of the striatum (Fig. 3B,C). Moreover, areas with increased levels of NAPEs, GM2, and GM3 matched those that had a decreased concentration of high-energy phosphate compounds (Supplementary Figure S7).

An apparent brain recovery was observed in two other HI-insulted rats. This finding was attributed to the biological variability in the rat pups exposed to moderate HI insult, particularly their exposure to hypoxia under mild hypothermia (34 °C). Modifying the original experimental model was important because the 36.5 °C conditions used previously resulted in a profound tissue reduction in the affected hemisphere and an overall mortality rate of 22% (data not shown). In the present study, this modification enabled us to follow the molecular changes in the HI-affected tissue, which still possessed sufficient integrity for the MSI application. Importantly, NAPEs, GM2, and GM3 were also present as endogenous molecules in the control brain; however, their distribution was uniform, and their concentration was markedly low (Supplementary Figure S7).

Similar to NAPEs, GM2 (d18:1/18:0) and GM3 (d18:1/18:0) were found in their deprotonated forms at *m/z* 1382.805 and *m/z* 1179.736, respectively, and were markedly increased in the HI-affected areas (Fig. 4). The GM2 ganglioside molecule was characterized according to the appropriate MS/MS fragmentation spectrum, showing characteristic neutral losses (Supplementary Figure S8). GM2 and GM3 showed a spatial distribution similar to that of NAPE species. Moreover, their population increased specifically in the area of the penumbra and spread to the insular cortex, piriform cortex, corpus callosum and external capsule.

**Figure 5:**
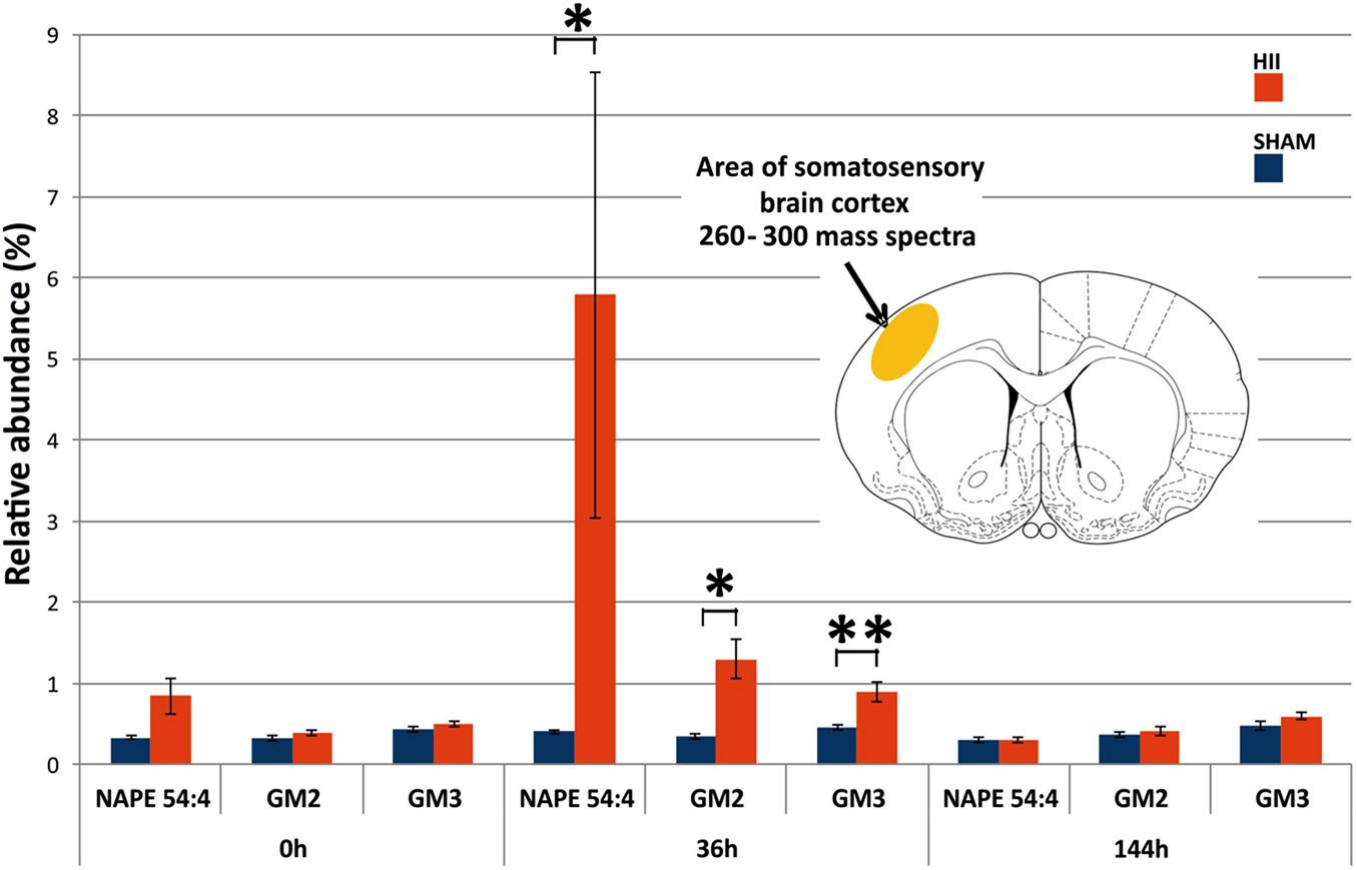
Relative abundance of the NAPE (54:4), GM2, and GM3 species in the sham (blue) and HI-affected (red) rat neonatal brains at 0 hour, 36 hours and 144 hours after the HI insult. Statistical significance of the measurement is displayed at the *p < 0.05 and **p < 0.01 levels.

The profiles of the most abundant NAPE representative (54:4), GM2 and GM3 are shown in Fig. 5. A somatosensory cortex area representing approximately 260–300 pixels was selected for the semi-quantitative statistical assessment, and the relative intensities of the [M-H]^−^ ions were normalized to PI (38:4), which represents the base peak at *m/z* 885 in all MALDI mass spectra. The statistical analysis of the differences between the HI and sham groups was performed using the non-parametric two-tailed Mann–Whitney U test. Statistical significance at a p-value < 0.05 (*p) was obtained for the sum of all NAPEs, GM2, and GM3. The same significance (*p < 0.05) was also obtained for NAPE (52:4), NAPE (54:4) and NAPE (58:6). Regarding GM3, the statistical significance was even higher, with a p-value equal to 0.004 (Supplementary Table S4). Regarding GM3, the statistical significance was also confirmed by performing a parametric two-tailed t-test, with a p-value equal to 0.016 (Supplementary Table S5). For the complete results, see the statistical analysis in the *Materials and methods* section.

Notably, in the semi-acute stage of the neonatal rat brain HI injury, we found remarkably increased NAPE, GM2 and GM3 levels in HI affected regions. In general, these compounds are important lipid mediators involved in early brain development and during injurious processes in the adult brain^19^. Our findings on the accumulation of NAPEs in the HI affected neonatal rat brain areas starting from 0 hour and peaking at 36 hours of the HI insult are in good agreement with the reports on markedly increased levels of NAPEs in the brain of adult rats and mice 5 and 24 hours after focal cerebral ischemia induction, respectively^20^. These data indicate involvement of NAPEs in the acute and semi-acute phase of HI brain injury. Importantly, NAPEs accumulation due to post-decapitative ischaemia was high in the brain of newborn rats, yet relatively low in adolescent and adult rats, indicating the significance of NAPEs in the developing brain^19^. NAPEs are precursors of *N*-acylethanolamines (NAEs), bioactive lipids with anti-inflammatory and neuroprotective effects, in particular protecting neurons from glutamatergic excitotoxicity^21^. Some of NAEs are endocannabinoids, e.g. anandamide (*N*-arachidonoylethanolamine), operating via the cannabinoid CB1/CB2 receptors and providing neuroprotection, including that from ischaemic damage^22,23^. In the present study, the increase of NAPEs in the affected brain areas observed at 36 hours of HI insult may reflect a tendency of the brain to limit the evolving HI injury.

Regarding the role of gangliosides in the developing brain, they are known to be synthesized and immediately metabolized in postnatal age. Our findings of low GM2/GM3 levels in the control brain of 7-day old rats are in agreement with the previous statement. Gangliosides were reported to accumulate during progressive neuronal damage^24^. Herein, we detected no increase in either GM2 or GM3 in the HI affected brain at 0 hour of the HI insult. On the other hand, their levels markedly increased at 36-hour time interval indicating the significance of GM2/GM3 in the semi-acute stage of neonatal brain HI injury. Whether the increased GM2/GM3 level reflects an effort of the HI brain to self-protect similarly to that of NAPE, this observation needs to be further elucidated.

### Compensation mechanism during the late stage after HI insult

We observed an ongoing restoration of the HI-affected brain tissue, and a gradual recovery of the overall brain architecture was observed on the sixth day (144 hours) after the HI insult. The cellular morphology, neurons and astrocytes in the HI-affected brain areas were not completely recovered as demonstrated by the distorted shape of the neuronal cells, the foamy appearance of the cytoplasm and signs of blobbing. The formation of a “halo” sign was observed around the neuronal cells. Angiogenesis, reflecting tissue compensation for the lack of oxygen, was observed. Despite these observed morphological changes, the neuronal cells had intact cellular membranes and preserved endoplasmic reticula (Supplementary Figure S9).

In contrast, considerable tissue loss was detected by MRI during the late stage of the insult. The overall tissue reduction in the HI-affected brains, as assessed by the ipsilateral/contralateral hemisphere volume ratio, was 13.1 ± 1.4% (mean ± standard error of the mean (SEM)) in a set of seven animals (Supplementary Table S6). In an analogous set of control animals (n = 6), the tissue loss was marginal. Similarly, no changes were observed between the HI-insulted and control sham rat brains at the molecular level using positive ion mode MALDI MSI measurements, while a slight persistently increased intensity of the monosialoganglioside GM3 was determined using the negative ion mode (four animals). No significant changes were observed in the levels of NAPEs or GM2 species. The multiple parametric and non-parametric testing of the time effect on the levels of NAPEs, GM2 and GM3 revealed statistically significant differences between times 0 and 36 hours for GM2 and GM3, and the respective p-values were 0.003 and 0.013 in the parametric tests and 0.046 and 0.039 in the non-parametric tests (see Supplementary Tables S7 and S8, respectively).

## Materials and Methods

### Chemicals and reagents

The isoflurane anaesthetic and nitrogen 4.0 were obtained from Baxter and Linde (both Bratislava, Slovak Republic), respectively. The indium-tin oxide (ITO) glass slides and α-cyano-4-hydroxycinnamic acid (CHCA) were obtained from Bruker Daltonics (Bremen, Germany). Acetonitrile, methanol, ethanol and water (all LC-MS grade) were obtained from Merck Millipore (Prague, Czech Republic). Trifluoroacetic acid (TFA), chloroform, sodium chloride, 9-aminoacridine (9-AA), haematoxylin solution, Gill no. 2 and DPX mounting media were obtained from Sigma-Aldrich (Prague, Czech Republic). Eosin Y (0.5%) aqueous solution was purchased from VWR Chemicals (Stribrna Skalice, Czech Republic). Hydrochloric and nitric acid (65%, Analpure) were obtained from FlukaBioChemika or Analytika Ltd., respectively (both Prague, Czech Republic). Deionized water (18.2 MΩ/cm) was prepared using a Milli-Q system (Millipore, Molsheim, France). Omni Slides with hydrophobic surfaces were purchased from Prosolia, Inc. (Zionsville, USA).

### Model of hypoxic-ischaemic brain injury in rat neonates

All animal experiments were conducted in accordance with the EU legislation (Directive 2010/63/EU), regulations and guidelines of the Slovak Animal Protection Act (Directive No. 377/2012 and Regulation No. 436/2012) under the approval of the State Veterinary and Food Administration of the Slovak Republic (Decision No. 2575/11–221/3) and the Animal Welfare Committee of the Institute of Experimental Pharmacology and Toxicology, CEM of the Slovak Academy of Sciences in Bratislava. Wistar rat females and males (both from the breeding station in Dobra Voda by Trnava, Slovak Republic) were mated at a 3:1 ratio for 6 days, separated and allowed to give birth at term. At the age of 7 days, the development and maturation of the rat brain are histologically similar to those in near-term human infants (32–36-week of gestation), and the typical characteristics include complete cortical neuronal layering, regression of germinal matrix and partial myelination^25^. Seven-day-old male pups (weighing 13–18 g) were anesthetized with isoflurane (4% for induction and 2% for maintenance), and the left common carotid artery was ligated with surgical silk^26^. Male pups were selected for the experiment because their sensitivity to hypoxic-ischaemic damage is known to be higher than that of female pups^27^. The neck incision was sutured, and the pups were allowed to recover from the anaesthesia for one hour. Subsequently, the pups were exposed to hypoxia (8% oxygen conditions, achieved by mixing air with gaseous nitrogen) in a sealed chamber at 34 °C for 90 minutes. The sham (control) animals were subjected to anaesthesia, surgical neck incision and common left carotid artery isolation but did not undergo ligation. After 1-hour recuperation, the sham pups were placed in a chamber with normoxic atmosphere at 34 °C for 90 minutes.

The overall experimental scheme consisted of two parallel experiments, i.e., *in vivo* and *ex vivo*. The brain changes at three time intervals after HI insult were assessed. In the *in vivo* experiments, the HI-affected (n = 9) and sham (n = 6) rat pups were allowed to survive, and MRI data were collected at 0-hour, 36-hour and 144-hour time intervals. Two HI-insulted rats did not survive to the sixth (144 h) day and were excluded from the experiment. In the *ex vivo* experiments, three sets of HI-affected and sham pups were sacrificed by decapitation at the 0-hour (n = 6/6, HI-affected/sham), 36-hour (n = 6/6) and 144-hour (n = 4/4) time intervals to assess the acute, subacute or late phase brain damage. All brains were quickly removed from the skull, snap-frozen in liquid nitrogen and stored at −80 °C until further analyses.

### Cryosectioning and histology

Prior to sectioning, the deeply frozen brain samples were allowed to warm at −11 °C in a Leica cryostat CM1950 (Wetzlar, Germany) chamber. The striatal level was located using a stereotaxic atlas^28^. In the MALDI MSI, LA-ICP-MSI and histology analyses, consecutive sections of 10-, 30- and 30-µm width were used, respectively. The sections were thaw-mounted onto precooled ITO glass slides for the MALDI MSI and light microscopy analyses. For the LA-ICP-MS analyses, the sections were directly deposited onto an Omni Slide without thawing. All slides were vacuum-dried in a desiccator at room temperature for 40 minutes prior to further use.

For histology evaluation, the slices were rehydrated in descending aqueous ethanol concentrations (100%, 95% and 80%) and stained with haematoxylin, Gill No. 2 (Sigma Aldrich, Prague, Czech Republic) to visualize the cellular nuclei. Then, the slide was flushed with water, and the excessive colorant was removed by acidic ethanol. Eosin counterstaining was subsequently performed. Tissue slices were serially dehydrated in ethanol solutions (95% and 100%), toned with xylene and permanently fixed with DPX mounting medium. Finally, the slides were examined as light-microscopic images using an Axio Scan.Z1 slide scanner (Zeiss, Jena, Germany) and processed using ZEN 2.3 Lite (Zeiss, Jena, Germany) software.

### MALDI mass spectrometry profiling and imaging

MALDI MS was performed using a 12 T SolariX FTICR mass spectrometer (Bruker Daltonics, Billerica, USA). CHCA (7 mg/mL in 50% ACN/0.1% TFA) and 9-AA (7 mg/mL in 75% MeOH) matrices were applied to the tissue attached onto the ITO glass slide using ImagePrep (Bruker Daltonics, Bremen, Germany, default method for CHCA). The product ion mass spectra were collected at 3 Da isolation width and a 30 V collision energy and processed using DataAnalysis 4.2 (Bruker Daltonics, Bremen, Germany). Desorption from the tissue was performed using laser attenuations of 22 or 26%. In the MS imaging experiments, the lipids and metabolites were ionized/desorbed using a 100-µm raster step size with a SmartBeam II laser (2 kHz, 200 shots/pixel) and 16,000 resolution (FWHM) at *m/z* 400. The raw MSI data were analysed using FlexImaging 4.1 (Bruker Daltonics, Bremen, Germany), transformed into an imzML file and processed using CycloBranch^29^.

### Quantitative laser ablation inductively coupled plasma mass spectrometry (LA-ICP-MS)

An Analyte G2 LA unit (Photon Machines, Redmont, USA) equipped with an ArF excimer nanosecond laser (193 nm) was used to perform the tissue ablation. The coupling of the LA and 7700× ICP-MS (Agilent Technologies, Tokyo, Japan) units was achieved with Tygon® (1.2 m × 4 mm) tubing. The raw data were imported as CSV files to ImageLab multisensor imaging software^30^, and the constructed distribution maps were correlated with the corresponding histological images. Details regarding the calibration plots and quantification are provided in Supplementary Figure S4.

The quantification experiments were performed using a set of dried droplet calibration standards as previously described^31,32^. Briefly, filter paper discs with a 5-mm diameter (Whatman 589/1, ashless) were placed onto a microscope glass slide using double-sided tape and spiked with 10 μL of a liquid standard solution with increasing concentrations of sodium and potassium (0, 10, 100, 500, 1000, and 4000 μg.mL^−1^ Na and K in 1% HNO_3_). Blank “calibration” was performed under the same conditions using 10 μL of 1% HNO_3_. After applying the liquids to the filter targets, the standards were allowed to dry at room temperature overnight in a Holten safe 2010 1.2 laminar flow cabinet (Live Technologies, Prague, Czech Republic).

### Magnetic resonance imaging

Each examined animal was placed onto a dedicated holder, and inhalation anaesthesia (2% isoflurane in air) was applied while the respiration rate was maintained within the 50–70 BPM range. All MRI experiments were performed at 37 °C using a 4.7 T direct-drive horizontal scanner (Agilent, Yarton, UK) equipped with a 400-mT/m gradient and 120-mm bore diameter insert. A Rapid Biomed (Rimpar, Germany) quadrature volume coil transmitter with an i.d. of 72 mm and a STARK Contrast (Erlangen, Germany) surface coil receiver were used for the signal detection. A diffusion-weighted sequence (FSEMSDW) was used with the following parameters: TR/TE_eff_ = 3000/36 ms, b-value = 1000 s/mm^2^, echo train length = 8, echo spacing = 10 ms, 10 averages, FOV = 25 × 25 mm^2^, and matrix size = 128 × 128. Eighteen contiguous 1-mm thick coronal slices were scanned to cover the brain regions of interest. The overall scan time was 8 min.

Both the data processing and volumetry were performed using ImageJ software, version 1.47^33^. A median filter with a 2-pixel radius was applied to reduce the noise and delineate the boundaries of the image. The overall lesion size was calculated by summing the hyperintensive area of all slices and multiplying by the slice thickness. Both the ipsilateral and contralateral lesion volumes were divided by the appropriate hemisphere volume. The measurement reliability was determined at the level of the slice for the oedema (n = 88) and brain (N = 108) measurements by evaluating the intra-class correlation coefficient using MedCalc for Windows, version 16.4 (MedCalc Software, Ostend, Belgium).

### Statistical analysis

The variability in the relative abundance of NAPEs, GM2, and GM3 in the technical replicates was measured by the coefficient of variation (CV), which is defined as the ratio of the standard deviation (SD) to the arithmetic mean (Supplementary Tables S9 and S10). The data are presented as the mean and SEM, and the differences between groups are presented as standard box-and-whisker diagrams with outliers plotted as individual points (Supplementary Figs S10–S16). The technical variability was less than 0.097 for NAPEs and GM2 and less than 0.135 for GM3. The analysis of variability demonstrated that the main component influencing the variability is biological variability. The percentage of biological variability in the total variability was within the ranges of 90.2–99.8%, 80.9–95.3%, and 39.1–93.2% for NAPEs, GM2, and GM3, respectively (Supplementary Table S11).

The biological variability was demonstrated by the coefficient of variation and variance decomposition using one-way ANOVA (Supplementary Table S7). The Gaussian distribution of the data sets was assessed by performing Lilliefors corrected Kolmogorov-Smirnov tests (Supplementary Table S12). The mean and SEM of the relative abundance of the NAPE representatives, GM2 and GM3 at the 0-, 36- and 144-hour time points are shown in Supplementary Table S13. The results of the normality testing were nonhomogeneous; thus, both parametric and non-parametric testing approaches were applied. The statistical significance of the differences between the mean values of the HI-insulted group and control group was tested by performing a two-tailed t-test (Supplementary Table S5). The equivalence test of medians was based on 95% confidence intervals calculated using a Mann–Whitney non-parametric U test between the HI-insulted and control groups (Supplementary Table S4). The time effect was determined using either an ordinary one-way ANOVA test (Supplementary Table S7) or a non-parametric Kruskal-Wallis test (Supplementary Table S8) and multiple comparison analysis testing. The significance level was set at p < 0.05, and all p-values were adjusted for multiple testing using the Bonferroni method. Individual rats in the HI-insulted group were compared with prediction intervals of expected values based on the control group (Supplementary Figs S17–S23).

### Data availability

All data generated or analysed in this study are included in this published article and its supplementary information files.

## Conclusions

In the present study, we simulated human neonatal HIE using a rat model and focused on the molecular and cellular processes in neonatal rat brains affected by HI insult. Acute stage cytotoxic oedema occurred as a result of cell swelling due to an influx of Na^+^, Cl^−^ and Ca^2+^, followed by water entry into intracellular compartments from the interstitial space. In contrast, the subacute stage vasogenic oedema, representing an extracellular type of oedema related to blood-brain barrier disruption, resulted from plasma protein extravasation, followed by water accumulation within the interstitial space of the brain^34^. We followed these two distinct processes using a multimodal imaging approach. Importantly, the elemental MSI detected a low Na^+^ abundance and high K^+^ abundance in the control brain slices, reflecting the normal intracellular distribution of these cations^35^. The acute response to HI insult involved a significant increase in the concentration of elemental Na^+^ and decrease in K^+^, which mirrored cell membrane depolarization. These respective alterations directly correlated with the observed changes in the profile of the Na^+^ and K^+^ adducts of the PCs as detected by MALDI MSI.

Based on the massive increase in NAPEs during the subacute phase, we speculate that their accumulation was the result of increased NAPE formation due to the degradation of the PI, PS and PA lipid species, whose intensities were decreased in the brain areas affected by HI injury. NAPE synthesis has been shown to increase under cellular stress and/or tissue damage and, as precursors of the *N*-acylethanolamine endocannabinoid ligands (NAE), NAPE may be involved in the endogenous neuroprotective reaction of the brain starting at the moment of an injurious process^36^. This finding correlates well with our results in which the accumulation of NAPEs started during the acute stage and then strongly increased due to progressive neurodegeneration during the subacute stage after the HI insult. Similarly to NAPE, we detected significantly higher intensities of GM2 and GM3 gangliosides in the affected brain regions compared to the non-affected areas and control brains. GM2 has been reported to accumulate along with the progressive destruction of neurons in a variety of neurological disorders^24^, including stroke and traumatic brain injuries^37,38^. The increased GM2/GM3 level may hence be considered as a marker of HI brain damage. Whether the observation of GM2 and GM3 just reflect injurious process, or these molecules are directly involved in the process of neuroprotection after the HI insult, both hypotheses still remain to be investigated^37,39^. To the best of our knowledge, there is just a single study that has reported alterations in brain gangliosides while using the same 7-day old rat experimental model of neonatal brain HI insult^40^. In that work an increased ganglioside GM3 level has been found in hippocampus of the exposed rat at 30 min and 1, 2 and 4 days after the HI insult. It is however difficult to compare those data with our findings due to substantially different tissue processing in the two studies. In the previous study^40^, an *ex vivo* processing of brain samples to get hippocampal homogenates, followed by laborious *in vitro* procedure and tissue extract analysis have been applied on the two hippocampal samples. In our study we applied *in vivo* fixation of the brain followed by *in situ* analysis of the entire lipidome in the whole brain section using mass spectrometry imaging techniques. Histopathological examination performed by us confirmed the significant impairment of the neuronal tissue, particularly in the cortical and striatum regions, which manifested as extensive liquefactive necrosis. Importantly, the most affected areas matched well with those of significantly increased NAPE, GM2 and GM3. Such increase of the particular lipid metabolites may reflect activation of neuroprotective mechanisms in the HI-insulted brain to minimize the damage.

Intriguingly, on the sixth day of HI injury, we observed a tendency of the neonatal rat brain to recover. We observed the disappearance of histopathological signs, and relatively intact neurons started to appear. These cells possessed morphological features that actually distinguished them from the control non-affected neurons. The concentration levels of neurodegenerative markers, such as NAPEs and GM2, decreased to nearly control levels, but a moderately increased level of GM3 was observed. According to the *in vivo* MR spectroscopy analysis of remarkably pronounced brain lesions in certain rats during the subacute stage of HI injury, animals that were severely affected by the insult are likely to suffer from adverse neurological outcomes. However, rats with apparently decreased brain lesions during the subacute stage subsequently showed recovery of the brain tissue. Overall, our findings are consistent with the fundamentals of current knowledge regarding this pathology, particularly its molecular and cellular processes, and should be considered in the development of novel therapeutic strategies for effective interventions in newborns affected by neonatal hypoxic-ischaemic encephalopathy. Along with modulating neurotrophin action by exogenous ganglioside analogues^41^, another way of neuroprotection and functional recovery of the damaged brain could involve (i) induction of endogenous NAPE formation and (ii) stimulation of NAPE transformation into neuroprotective NAEs, including those modulating CB receptors.

## Electronic supplementary material

Supplementary file
