# Supplementary material for: Membrane depolarization and aberrant lipid distributions in the neonatal rat brain following hypoxic-ischaemic insult
Source: Sci Rep. 2018 May 3;8:6952. doi: 10.1038/s41598-018-25088-2 (PMC5934395; doi:10.1038/s41598-018-25088-2)
Supplement: Supplementary file 1 — Supplementary file [file 41598_2018_25088_MOESM1_ESM.pdf]

SUPPLEMENTARY FILE

Membrane depolarization and aberrant lipid distributions in the neonatal rat brain following hypoxic-ischaemic insult

Dominika Luptakova[1,2], Ladislav Baciak[2,3], Tomas Pluhacek[1,4], Anton Skriba[1], Blanka Sediva[1], Vladimir Havlicek[1,4*], Ivo Juranek[2]

[1]Institute of Microbiology of the Czech Academy of Sciences, Prague 4, 142 20, Czech Republic

[2]Institute of Experimental Pharmacology and Toxicology, CEM of the SAS, Bratislava, 841 04, Slovakia

[3]Slovak University of Technology, Central Laboratories, Bratislava, 812 37, Slovakia

[4]Regional Centre of Advanced Technologies and Materials, Department of Analytical Chemistry, Olomouc, 771 47, Czech Republic

[*]vlhavlic@biomed.cas.cz

Table of Contents:

Figure S1: Acute (cytotoxic) brain ooedema evolution in 7-day old rat pup (**panel A;** *in vivo* DW-MRI image taken at 0 h after HI insult of pup #16). **Panel B** – Volume of acute eodema in the ipsilateral (ipsi, dotted line) and contralateral (contra, full line) hemisphere (mean ± SEM, n = 7). **Panel C** – Evolution of the total lesion size relative to the whole brain volume in time; two rats (out of nine) that died around 36-hour interval were excluded from the graph. Rats # 3, 5, 7, 10 and 18 recovered in time (green lines) while rats # 9 and 16 developed progressive aggravation of brain lesion (red lines). **Panel D** – Representative coronary brain MRI consecutive scans (N=8) of pup #9 taken at acute (0 h), subacute (36 h) and late phase (144 h) of HI injury; cytotoxic oedema is indicated by red arrows (0 h); vasogenic oedema/early necrosis reflected by hypointensive MRI signal are indicated by white arrows (36 h, 144 h).

Figure S2: Representative averaged MALDI mass spectra of affected rat pup brain tissue acquired in positive ion mode (**A**) and negative ion mode (**B**).

Figure S3: MALDI MSI analysis showing the spatial distribution of Na^+ and K^+ adducts (upper and lower panel, respectively) of phosphocholine PC (14:0/16:0) within the representative HI-insulted (HII) and control (sham) brain at striatal level; the respective characteristic fragmentation spectra are depicted on the left.

Figure S4: Sodium and potassium calibration plots in LA-ICP-MSI. Two line scans were ablated along the whole diameter of each filter paper disks (no tissue matrix was present) in the following order: 0, 10, 100, 500, 1000 and 4000 $\mu g.g^{-1}$ for both sodium and potassium. The background-subtracted signal intensity data were averaged over the two line scans per each concentration level and plotted against the spiked concentrations. For both calibration curves, the linear relationship was found with the coefficient of determination value over 0.994. Sodium and potassium signals were divided by the

corresponding ^{12}C isotope signal. This standardization allowed for signal compensation arising from different interactions of the laser with the sample surface as well as local differences in the tissue thickness.

Figure S5: The fragmentation spectrum of adenosine diphosphate (ADP) in a negative ion mode (MALDI).

Figure S6: The fragmentation spectrum of adenosine monophosphate (AMP) in a negative ion mode (MALDI).

Figure S7: MALDI MSI analysis of the subacute molecular changes in the HI-affected rat pup brain (36 h after the insult); molecules discussed in the main text were: phosphocholines (PC), adenosine monophosphate (AMP), adenosine diphosphate (ADP), *N*-acylphosphatidylethanolamine (NAPE), gangliosides (GM2 and GM3), phosphoserines (PS), phosphatidylinositols (PI) and phosphoethanolamines (PE).

Figure S8: The fragmentation spectrum of monosialoganglioside GM2 in a negative ion mode (MALDI). All product ions arose from the dissociation in saccharidic part of the molecule.

Figure S9: Typical histological picture of the rat pup brain at the late phase (144 h) of HI injury (Panels A, C, D) in comparison to the sham brain (panel B). In somatosensory cortex, morphologically deformed neurons (red circles) were observed in the HI-insulted (ipsilateral) hemisphere (A) while intact neurons (green circle) prevailed in the sham brain (B). At striatal level, necrotic areas (marked in brown) were observed (C); individual necrotic cells (white circles) and reactive astrocyte (black circle) seen in enlarged image (D).

Figures S10-S16: Graphical representation of differences in means and medians between HI-insulted (HII) group and control group using standard box-and-whisker diagrams.

Figures S17-S23: Graphical representation of the individual results for rats of the HII group compared with the 95% prediction intervals of expected value based on control group (red area – prediction intervals based on control group)

Table S1: Lesion size related to cytotoxic ooedema expressed as hemisphere (ipsilateral/contralateral) volume in seven experimental rats. SD and SEM stand for Standard Deviation and Standard Error of the Mean, respectively.

Table S2: Characterization of the observed Na^+ and K^+ adducts of phosphocholines (PCs).

Table S3: *N*-acylphosphatidylethanolamines (NAPEs) detected in the HI-affected brains at 36 h after the HI insult.

Table S4: Statistical analysis of differences between the HII group and control group performed using nonparametric two-tailed Mann–Whitney U test. *P* values indicating statistical significance are in red color.

Table S5: Statistical analysis of differences between the HII group and control group performed using parametric two-tailed t-tests. *P* values indicating statistical significance are in red color.

Table S6: Tissue reduction at 144 hours after the HI insult recorded by MRI.

Table S7: Multiple parametric testing of the time effect based on one-way ANOVA. *P* values indicating statistical significance are in red color.

Table S8: Multiple nonparametric testing of the time effect based on Kruskal-Wallis method. *P* values indicating statistical significance are in red color.

Table S9: Technical variability expressed as Coefficients of Variation (CV) for each group of technical replications.

Table S10: Biological variability expressed as Coefficient of Variation (CV).

Table S11: Percentage of the biological variability from the total variability for each dataset.

Table S12: *P*-values of the Lilliefors corrected Kolmogorov-Smirnov test of the normality. Values indicating statistical significance (if the *P*-value rejected normal distribution) are in red.

Table S13: Values (mean ± SEM) of the relative abundance of the NAPE representatives, GM2 and GM3 of the 0, 36 and 144 time intervals.

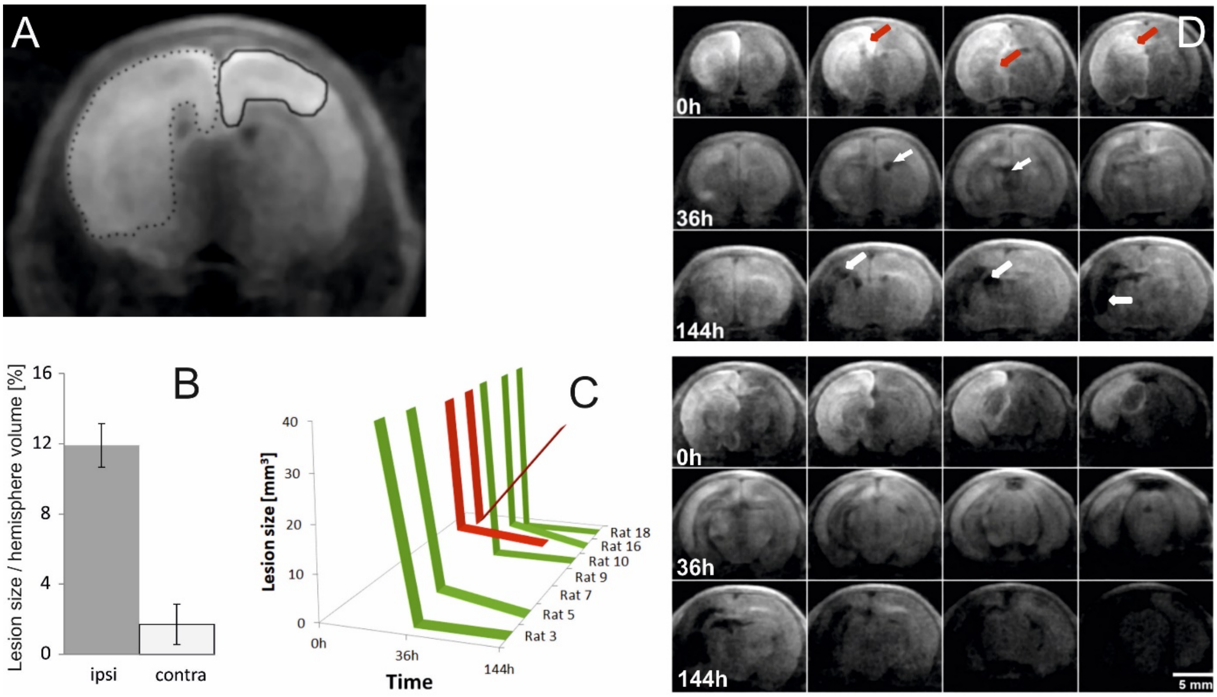

Figure S1: Acute (cytotoxic) brain oedema evolution in 7-day old rat pup (**panel A;** *in vivo* DW-MRI image taken at 0 h after HI insult of pup #16). **Panel B** – Volume of acute oedema in the ipsilateral (ipsi, dotted line) and contralateral (contra, full line) hemisphere (mean ± SEM, n = 7). **Panel C** – Evolution of the total lesion size relative to the whole brain volume in time; two rats (out of nine) that died around 36-hour interval were excluded from the graph. Pups # 3, 5, 7, 10 and 18 recovered in time (green lines) while pups # 9 and 16 developed progressive aggravation of brain lesion (red lines). **Panel D** – Representative coronary brain MRI consecutive scans (N=8) of pup #9 taken at acute (0 h), subacute (36 h) and late phase (144 h) of HI injury; cytotoxic oedema is indicated by red arrows (0 h); vasogenic oedema/early necrosis reflected by hypointensive MRI signal are indicated by white arrows (36 h, 144 h).

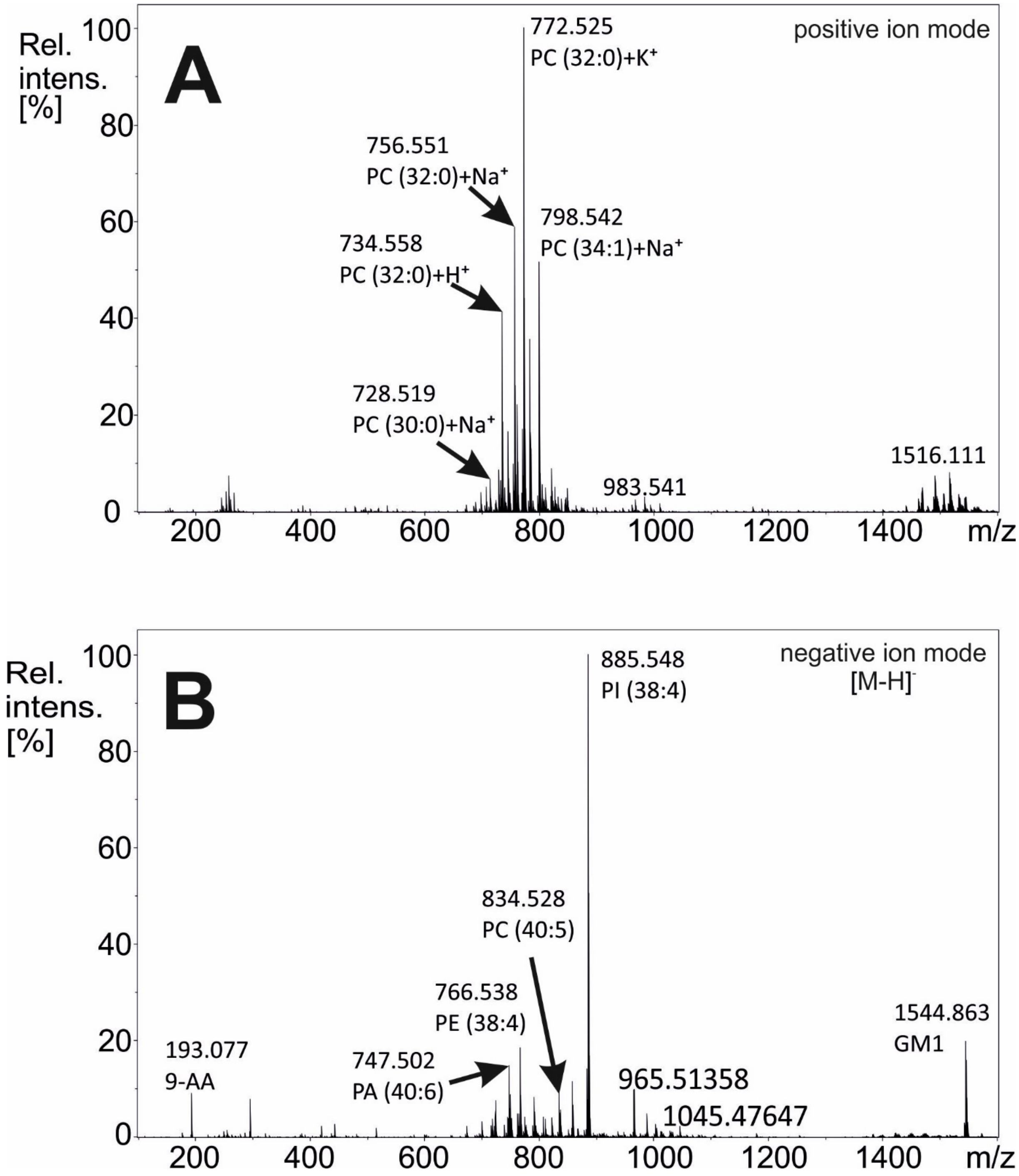

Figure S2: Representative averaged MALDI mass spectra of affected rat pup brain tissue acquired in positive ion mode (**A**) and negative ion mode (**B**).

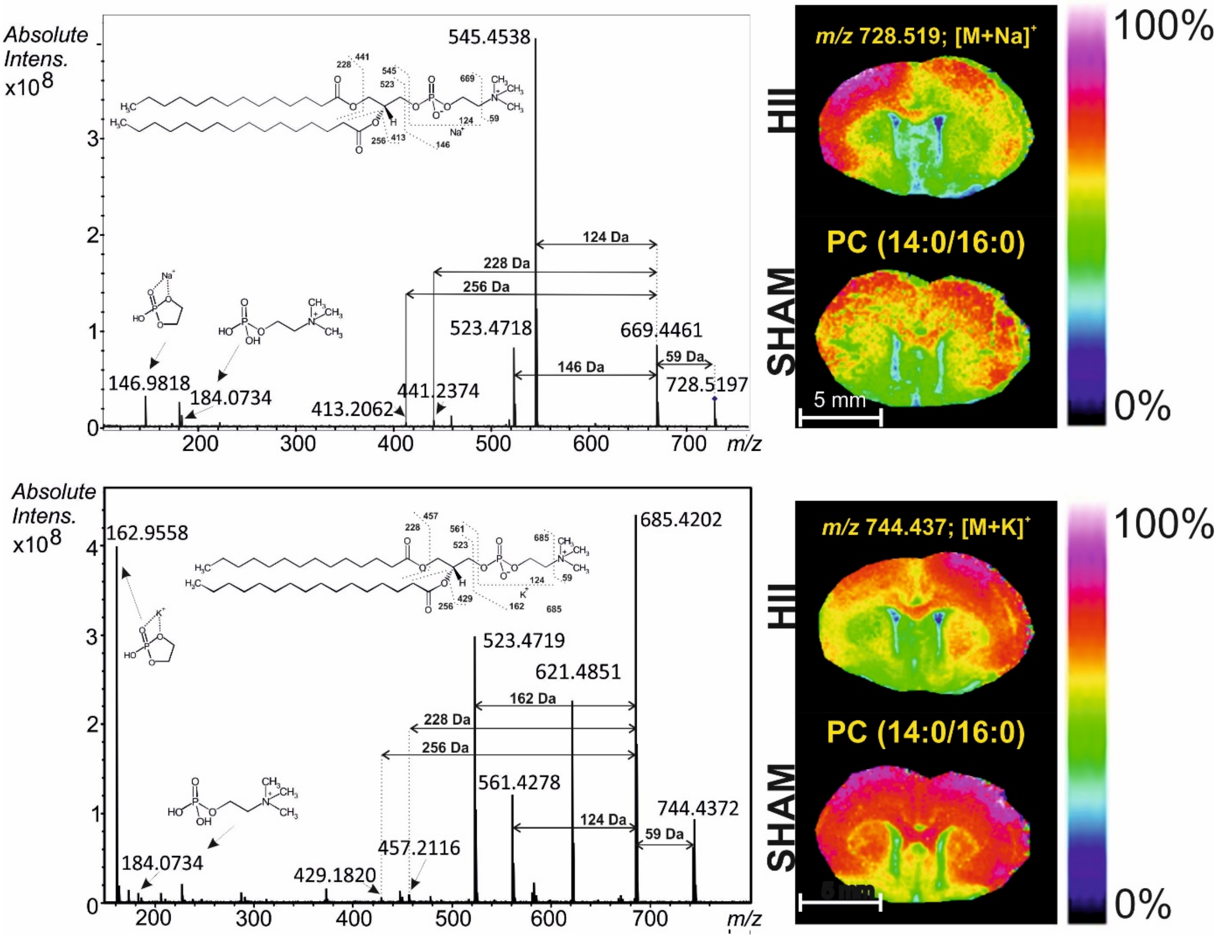

Figure S3: MALDI MSI analysis showing the spatial distribution of Na^+ and K^+ adducts (upper and lower panel, respectively) of phosphocholine PC (14:0/16:0) within the representative HI-insulted (HII) and control (sham) brain at striatal level; the respective characteristic fragmentation spectra are depicted on the left.

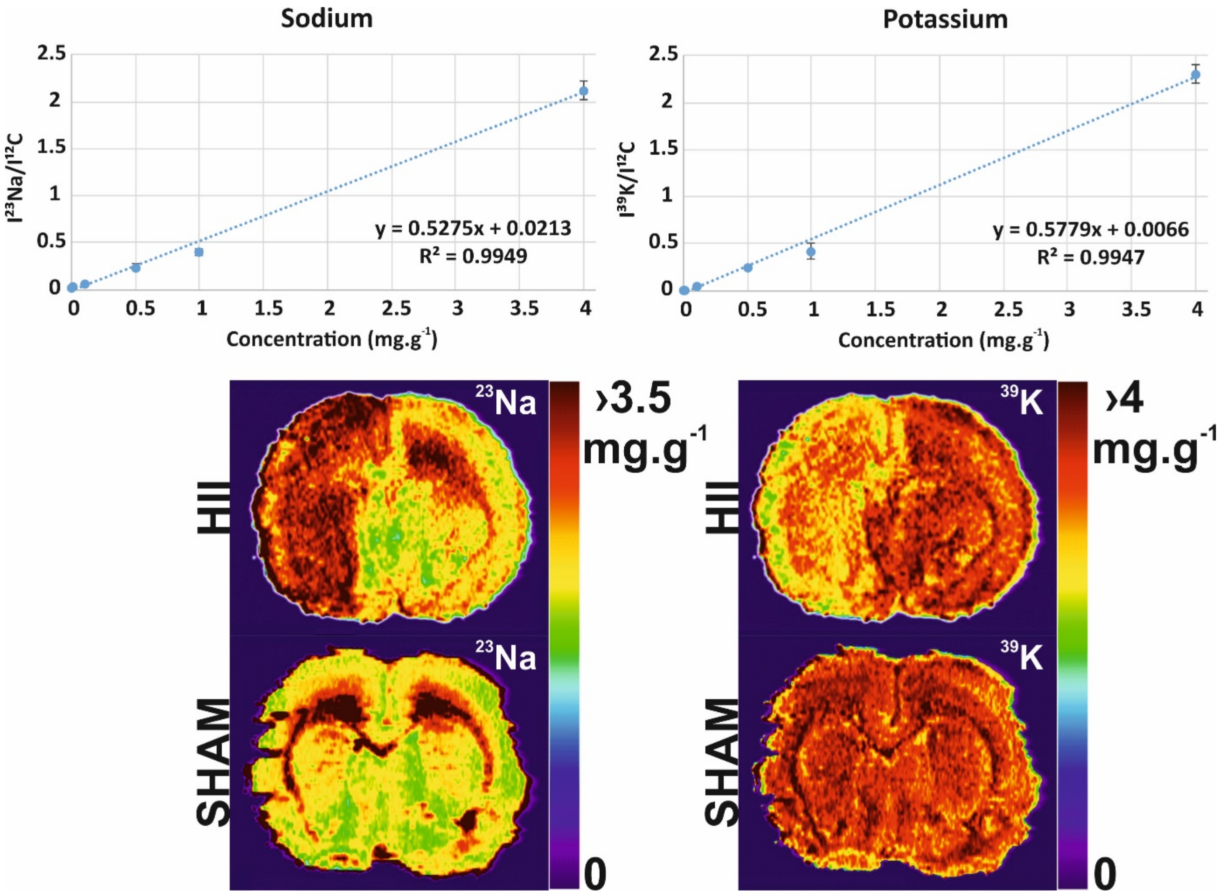

Figure S4: Sodium and potassium calibration plots in LA-ICP-MSI. Two line scans were ablated along the whole diameter of each filter paper disks (no tissue matrix was present) in the following order: 0, 10, 100, 500, 1000 and 4000 µg.g^{-1} for both sodium and potassium. The background-subtracted signal intensity data were averaged over the two line scans per each concentration level and plotted against the spiked concentrations. For both calibration curves, the linear relationship was found with the coefficient of determination value over 0.994. Sodium and potassium signals were divided by the corresponding ^{12}C isotope signal. This standardization allowed for signal compensation arising from different interactions of the laser with the sample surface as well as local differences in the tissue thickness.

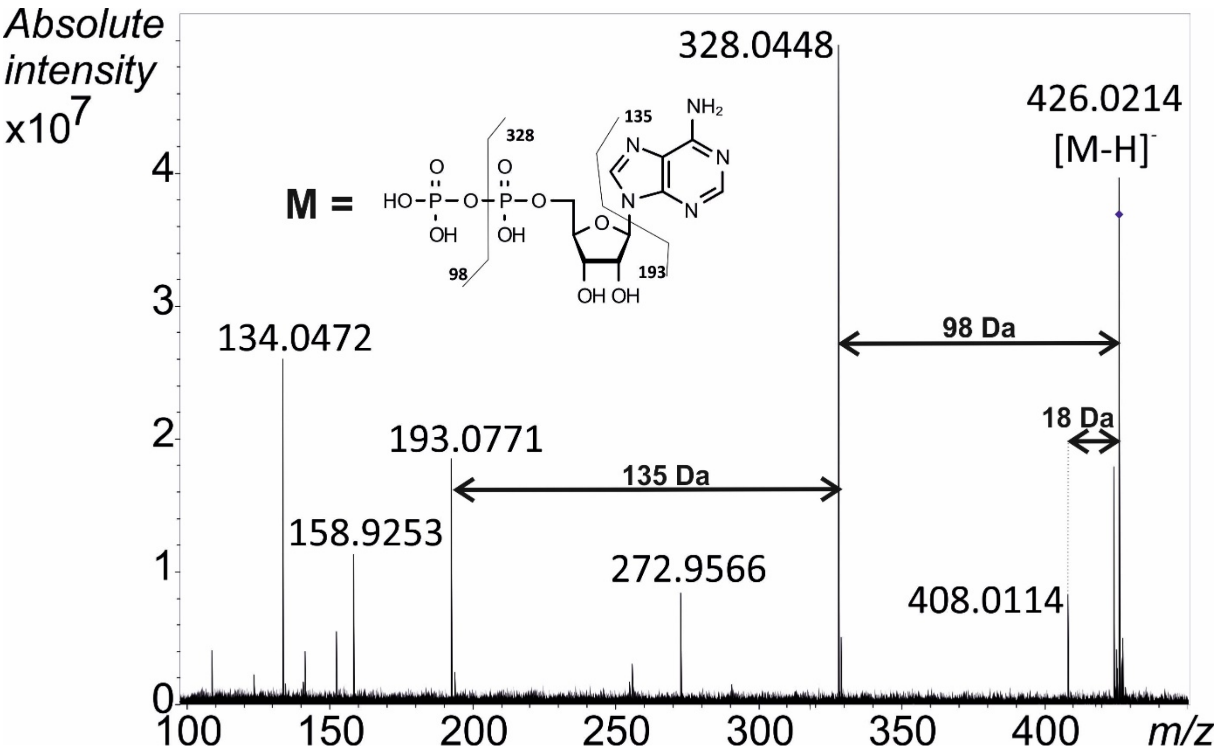

Figure S5: The fragmentation spectrum of adenosine diphosphate (ADP) in a negative ion mode (MALDI).

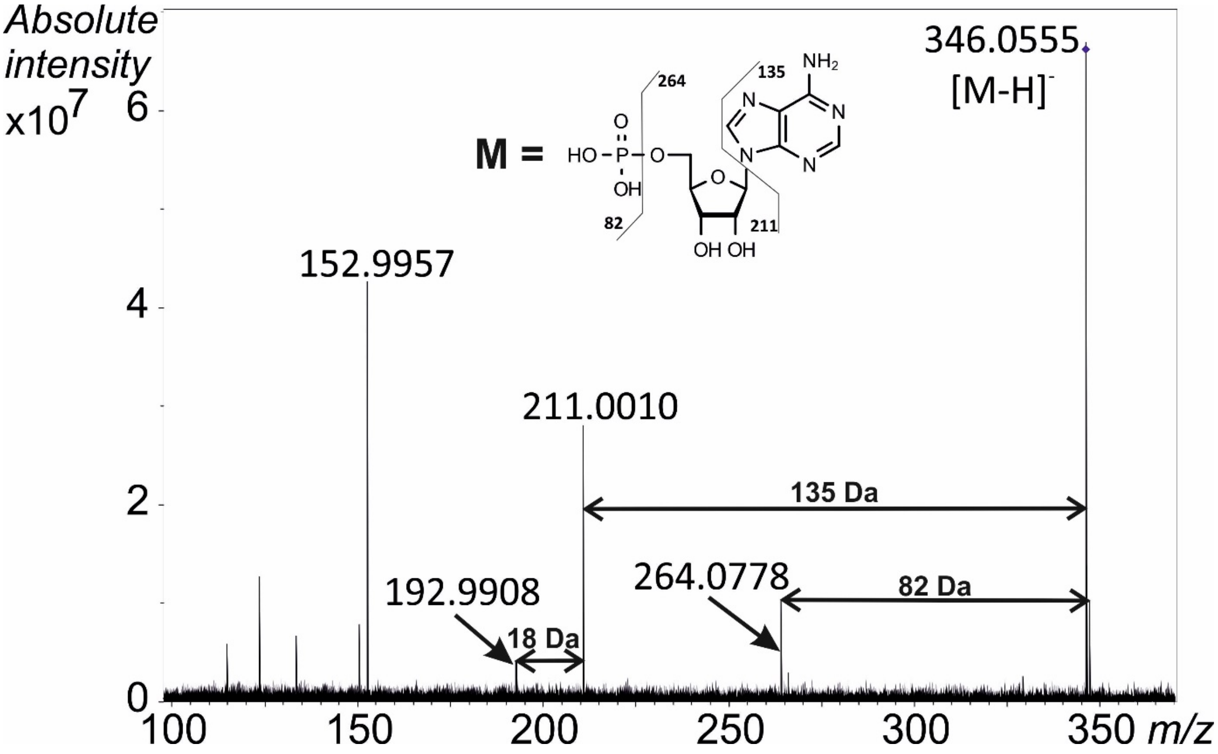

Figure S6: The fragmentation spectrum of adenosine monophosphate (AMP) in a negative ion mode (MALDI).

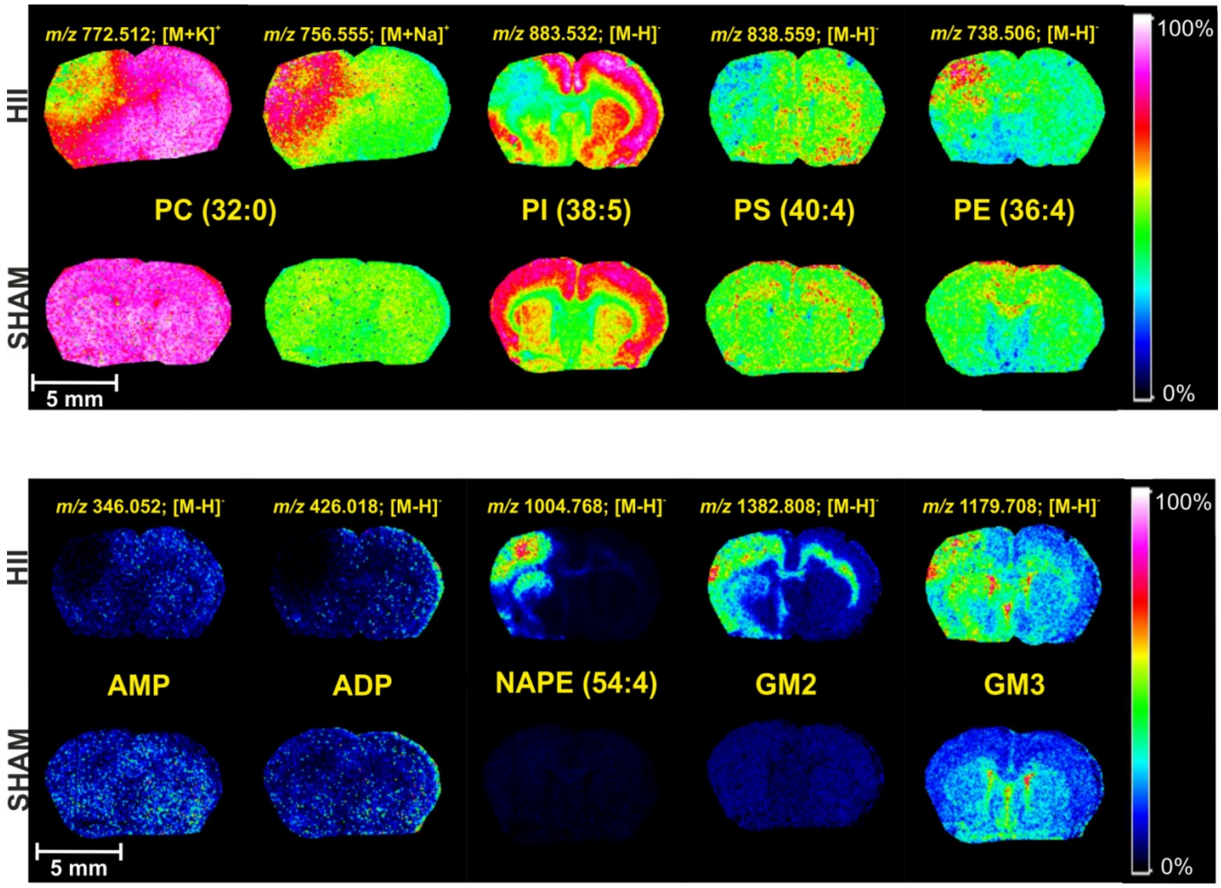

Figure S7: MALDI MSI analysis of the subacute molecular changes in the HI-affected rat pup brain (36 h after the insult); molecules discussed in the main text were: phosphocholines (PC), adenosine monophosphate (AMP), adenosine diphosphate (ADP), *N*-acylphosphatidylethanolamine (NAPE), gangliosides (GM2 and GM3), phosphoserines (PS), phosphatidylinositols (PI) and phosphoethanolamines (PE).

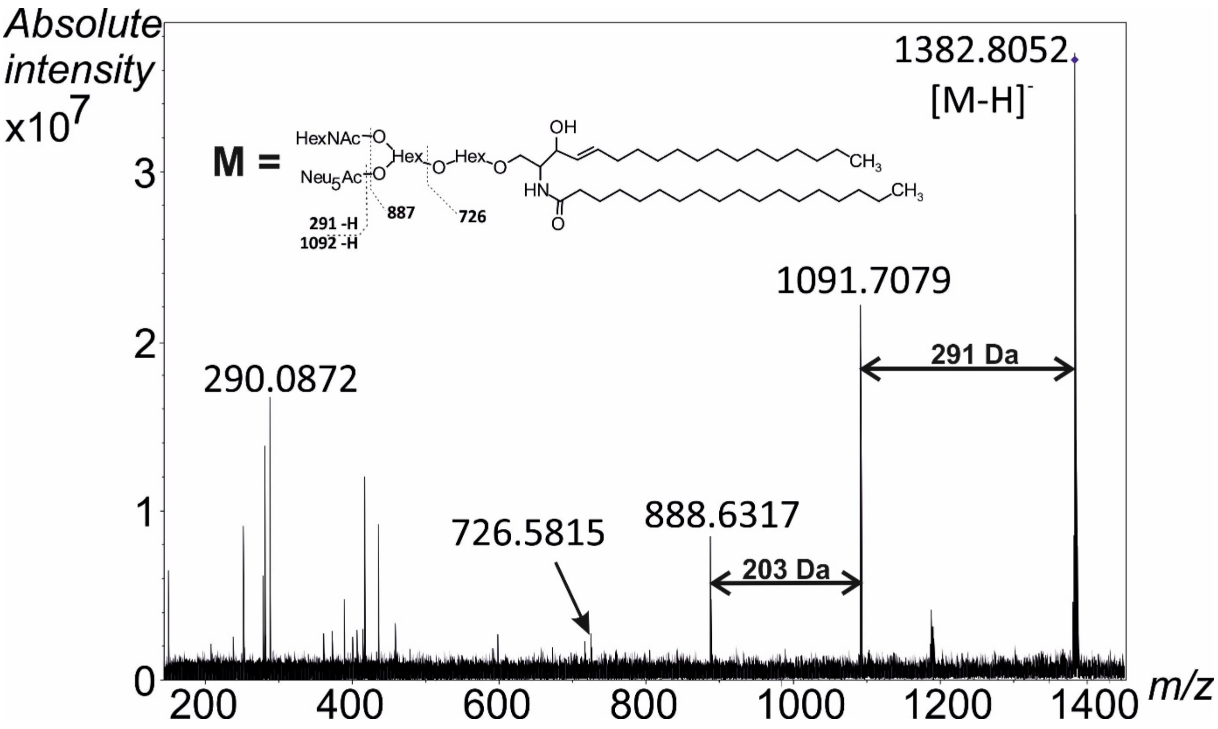

Figure S8: The fragmentation spectrum of monosialoganglioside GM2 in a negative ion mode (MALDI). All product ions arose from the dissociation in saccharidic part of the molecule.

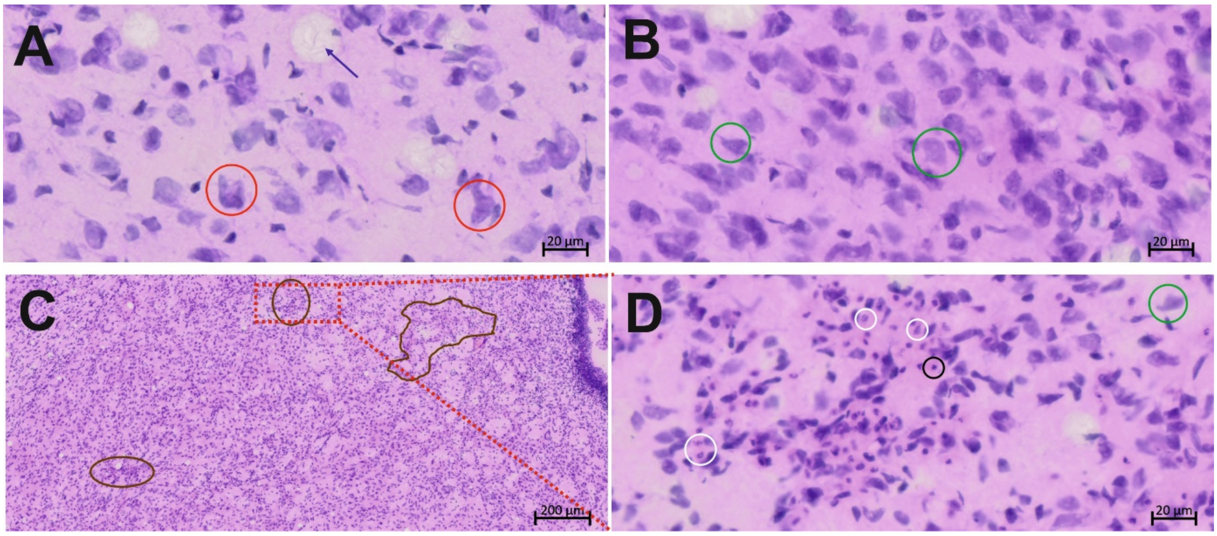

Figure S9: Typical histological picture of the rat pup brain at the late phase (144 h) of HI injury (**Panels A, C, D**) in comparison to the sham brain (**panel B**). In somatosensory cortex, morphologically deformed neurons (red circles) were observed in the HI-insulted (ipsilateral) hemisphere (**A**) while intact neurons (green circle) prevailed in the sham brain (**B**). At striatal level, necrotic areas (marked in brown) were observed (**C**); individual necrotic cells (white circles) and reactive astrocyte (black circle) seen in enlarged image (**D**).

Supplemental Figures S10-S16: Graphical representation of differences in means and medians between HI-insulted (HII) group and control group using standard box-and-whisker diagrams.

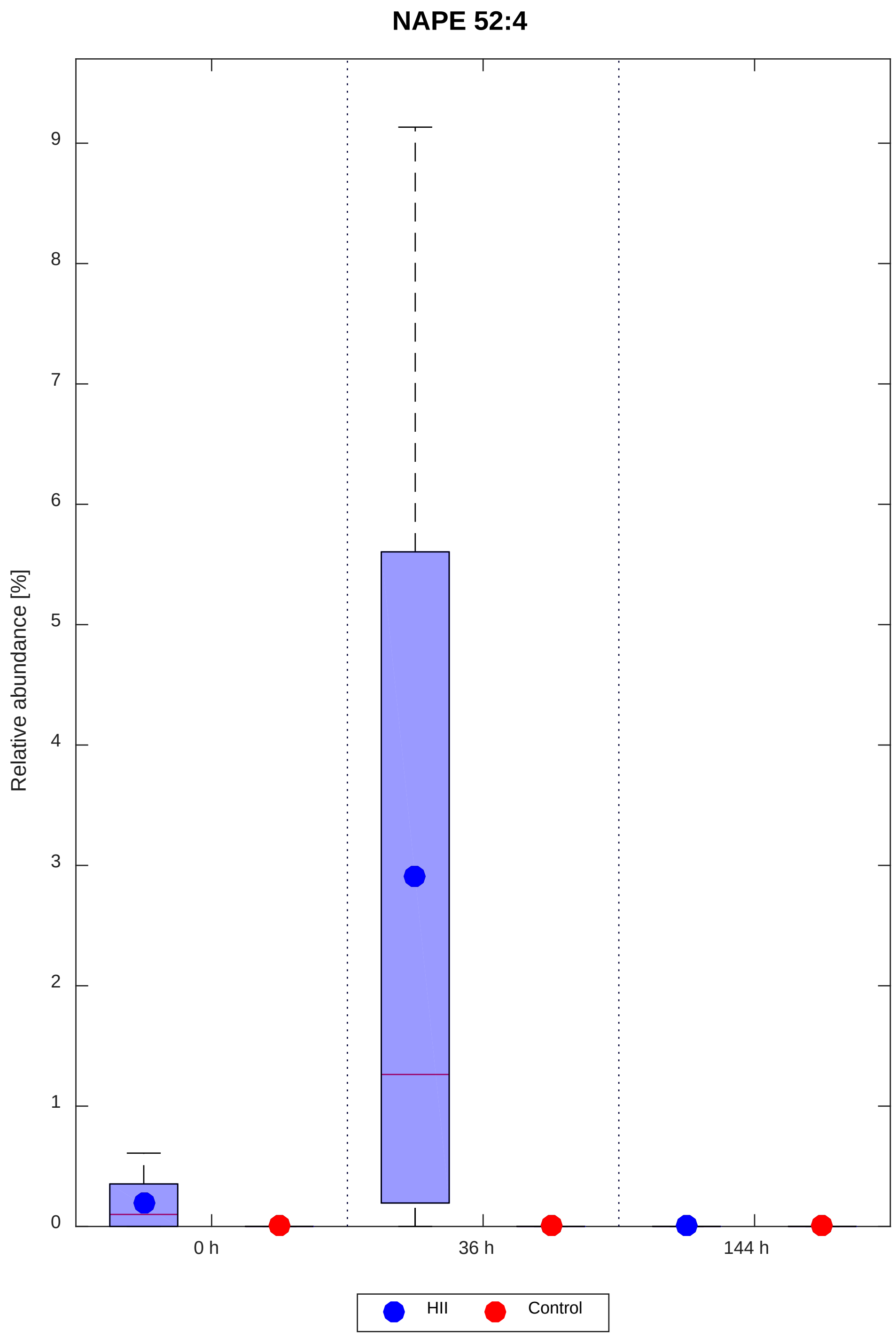

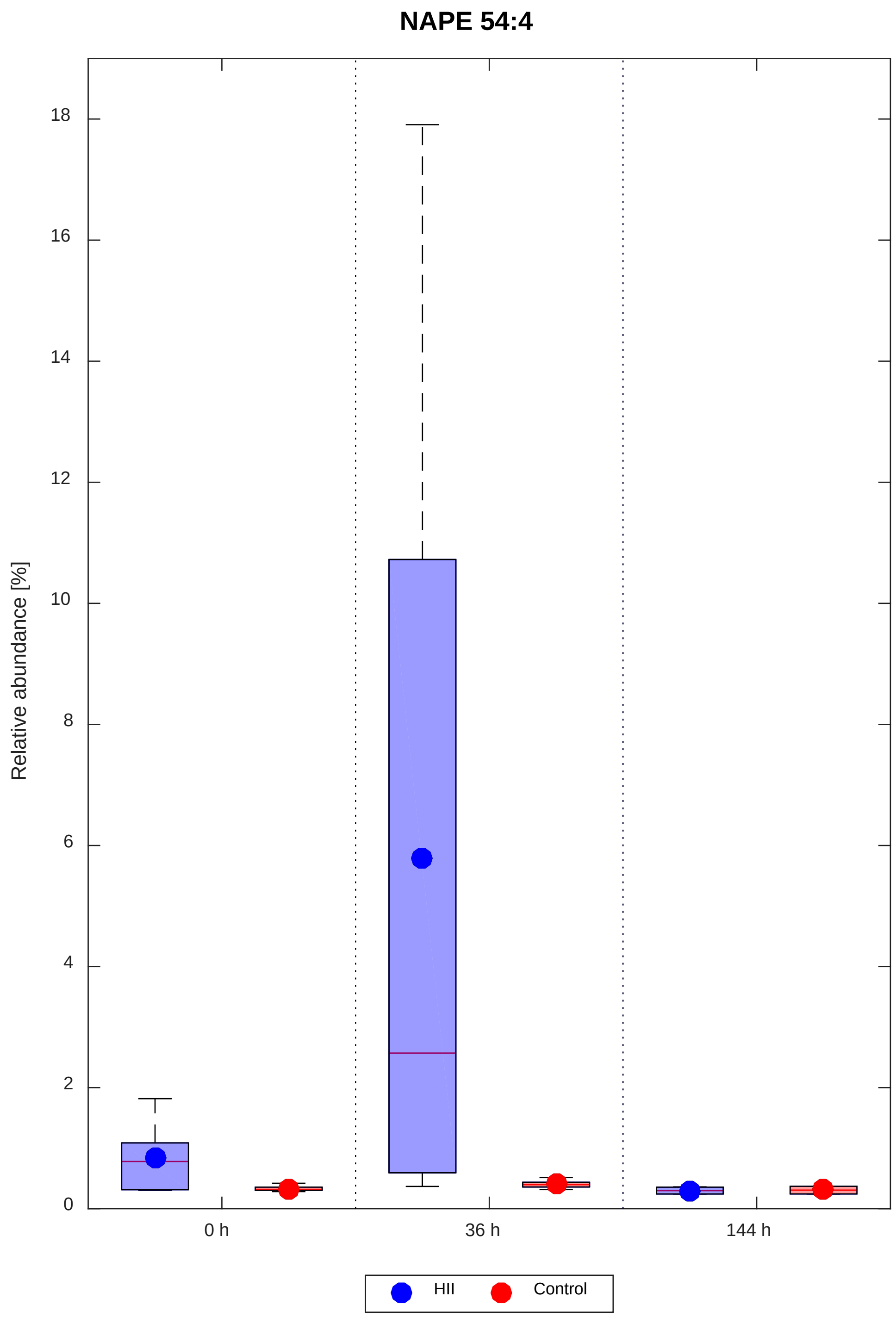
NAPE 54:4
Relative abundance [%]
0
2
4
6
8
10
12
14
16
18
0 h
36 h
144 h
HII
Control

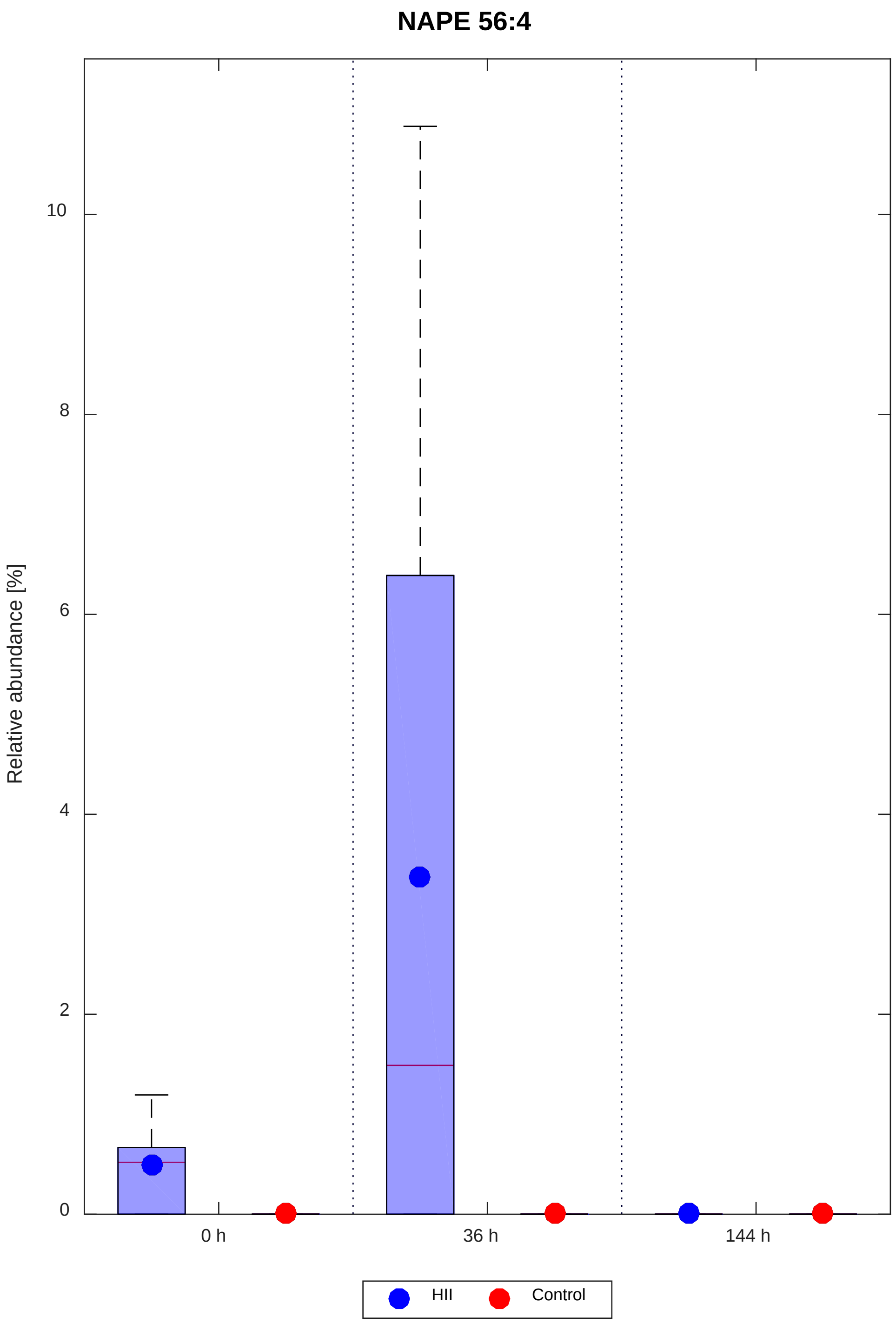
NAPE 56:4
Relative abundance [%]
0
2
4
6
8
10
0 h
36 h
144 h
HII
Control

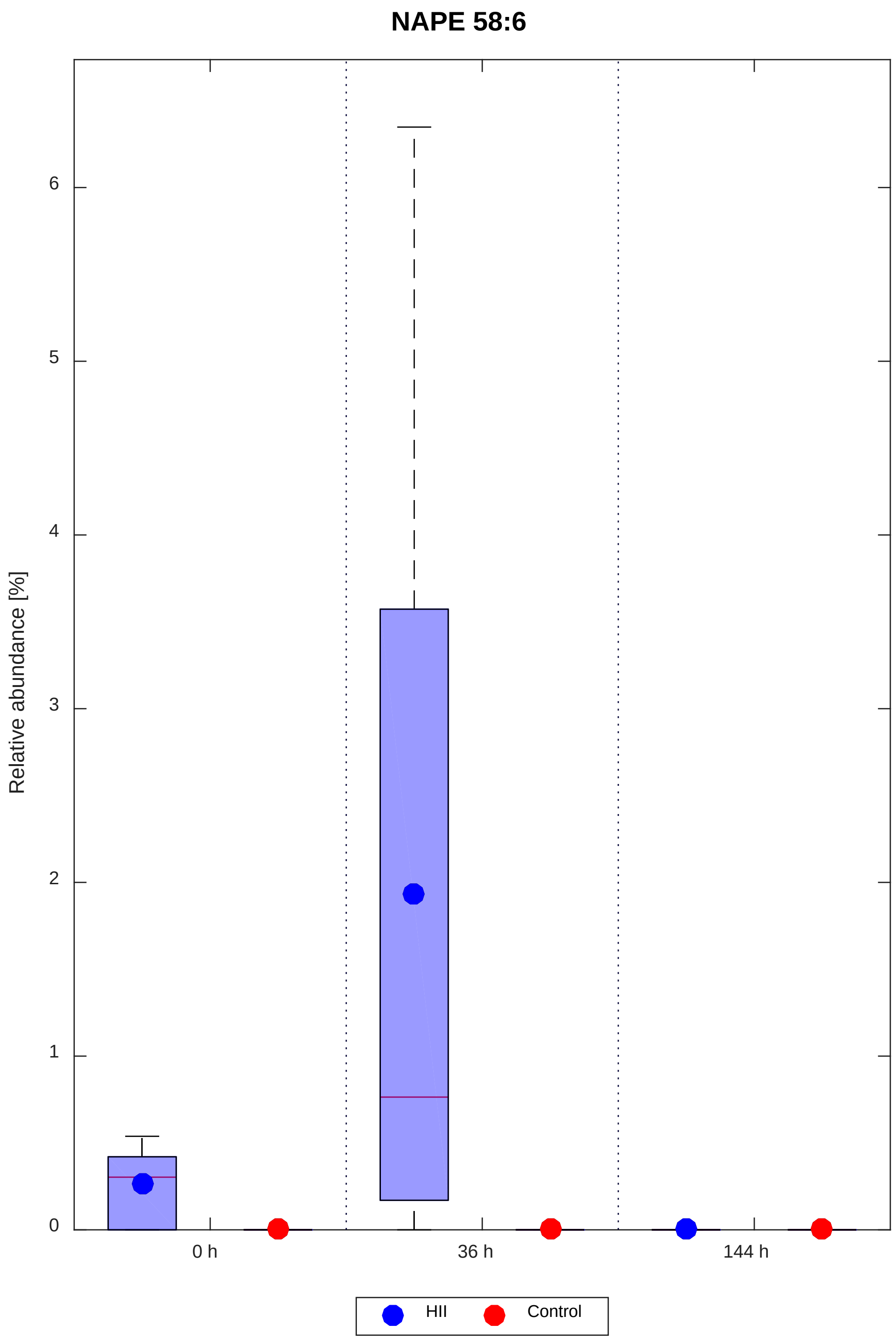
NAPE 58:6
Relative abundance [%]
0
1
2
3
4
5
6
0 h
36 h
144 h
HII
Control

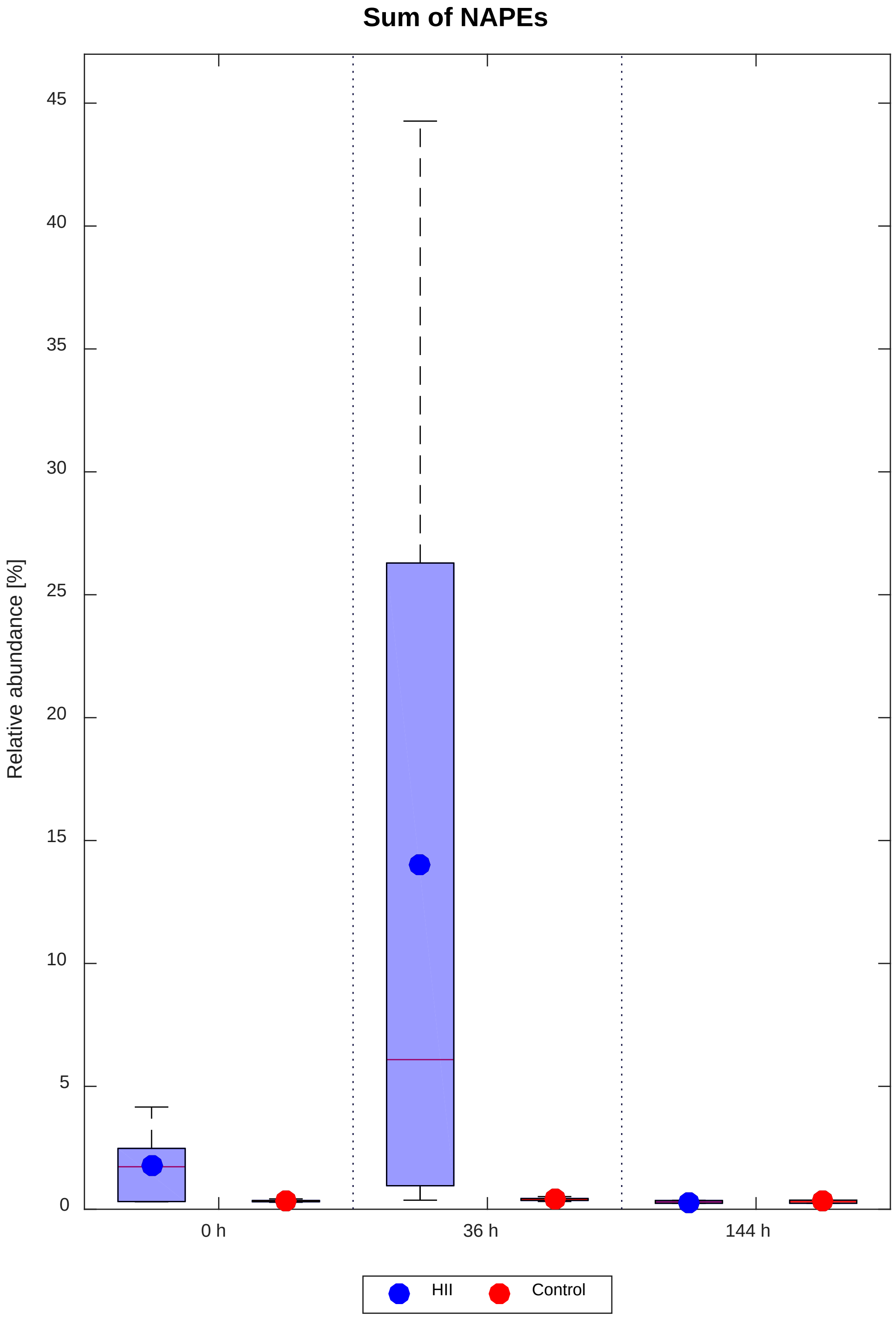
Sum of NAPEs
Relative abundance [%]
45
40
35
30
25
20
15
10
5
0
0 h
36 h
144 h
HII
Control

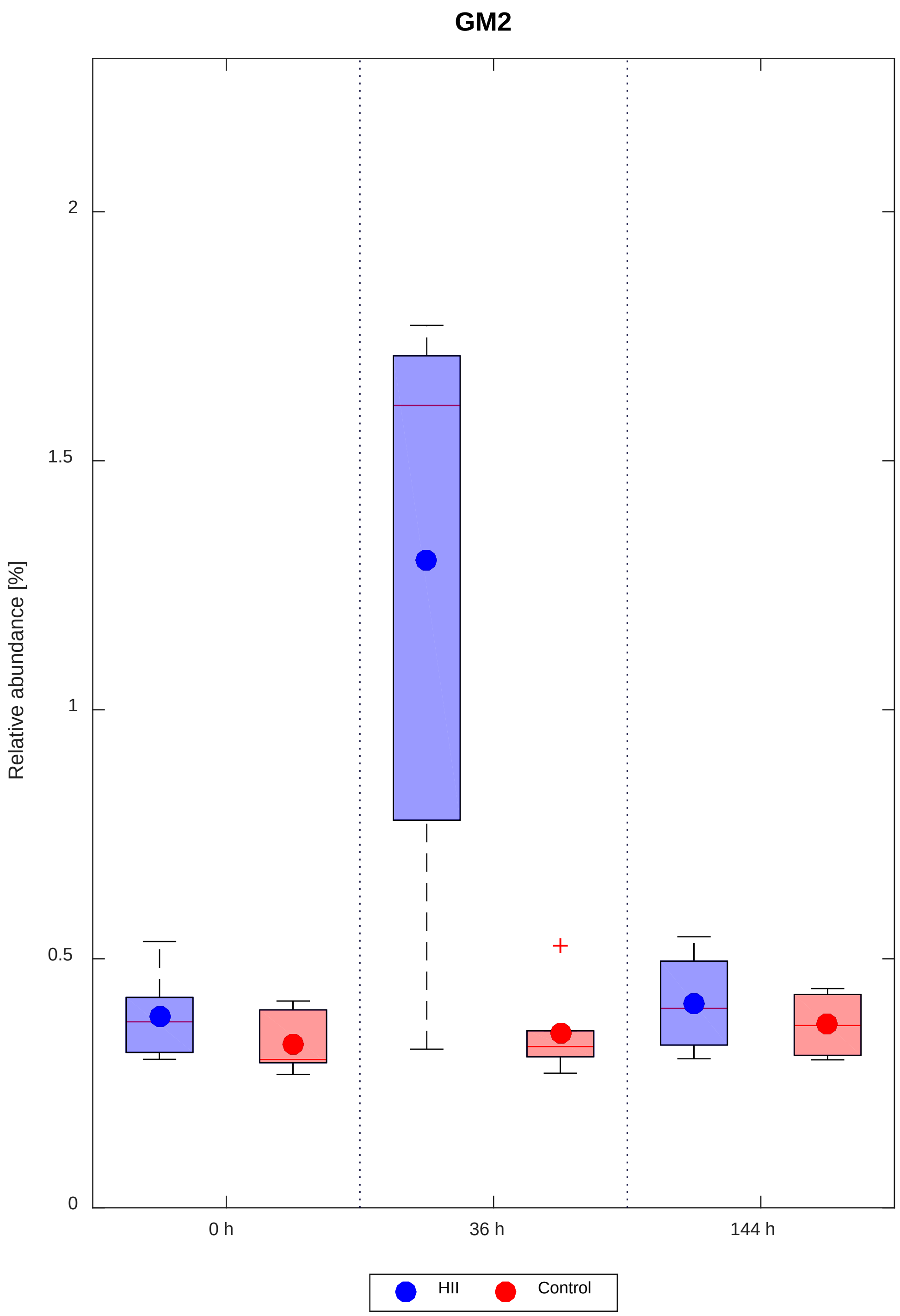
GM2
Relative abundance [%]
0
0.5
1
1.5
2
0 h
36 h
144 h
HII
Control

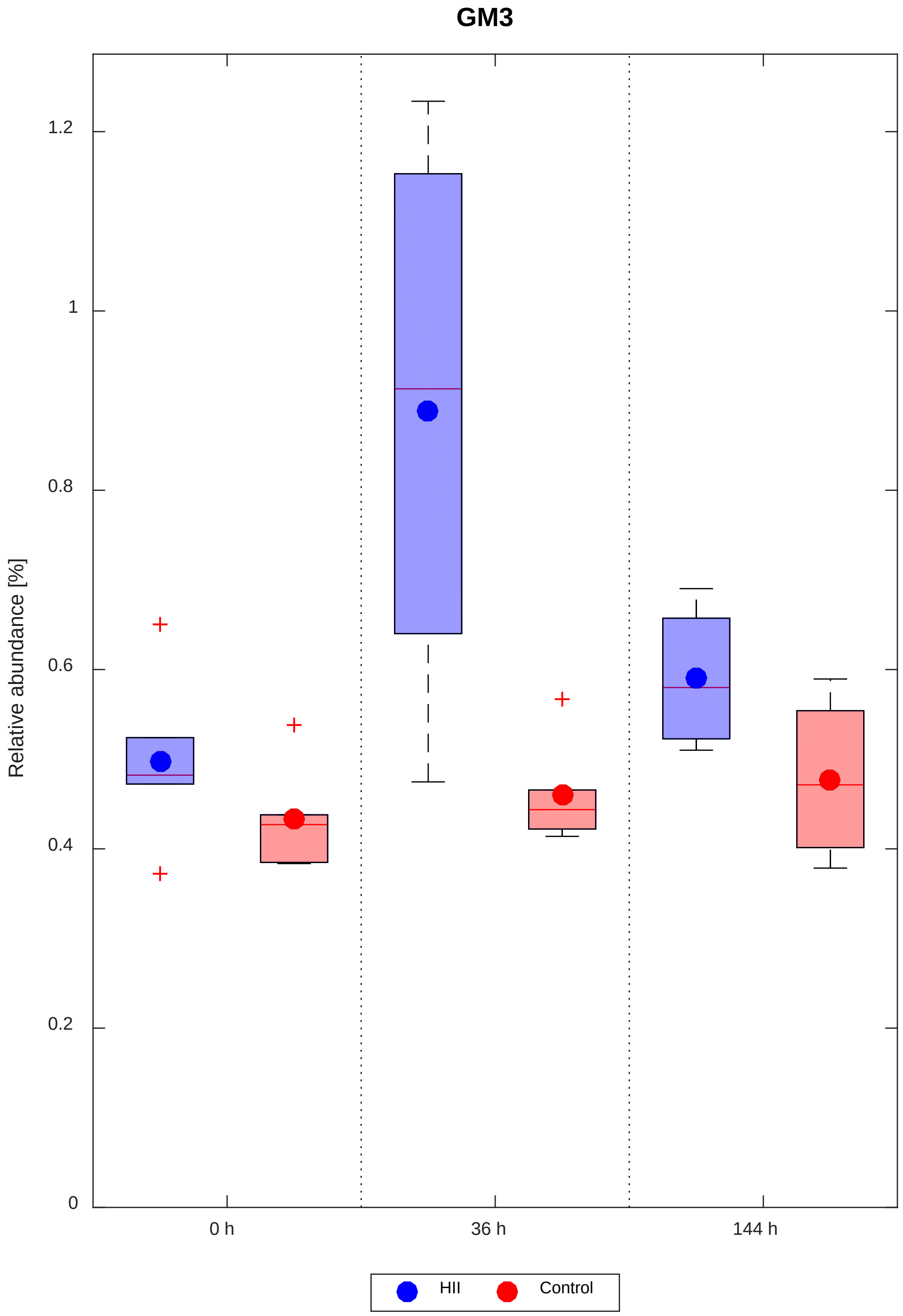
GM3
Relative abundance [%]
0
0.2
0.4
0.6
0.8
1
1.2
0 h
36 h
144 h
HII
Control

Supplemental Fig S17-S23: Graphical representation of the individual results for rats of the HII group compared with the 95% prediction intervals of expected value based on control group (red area – prediction intervals based on control group)

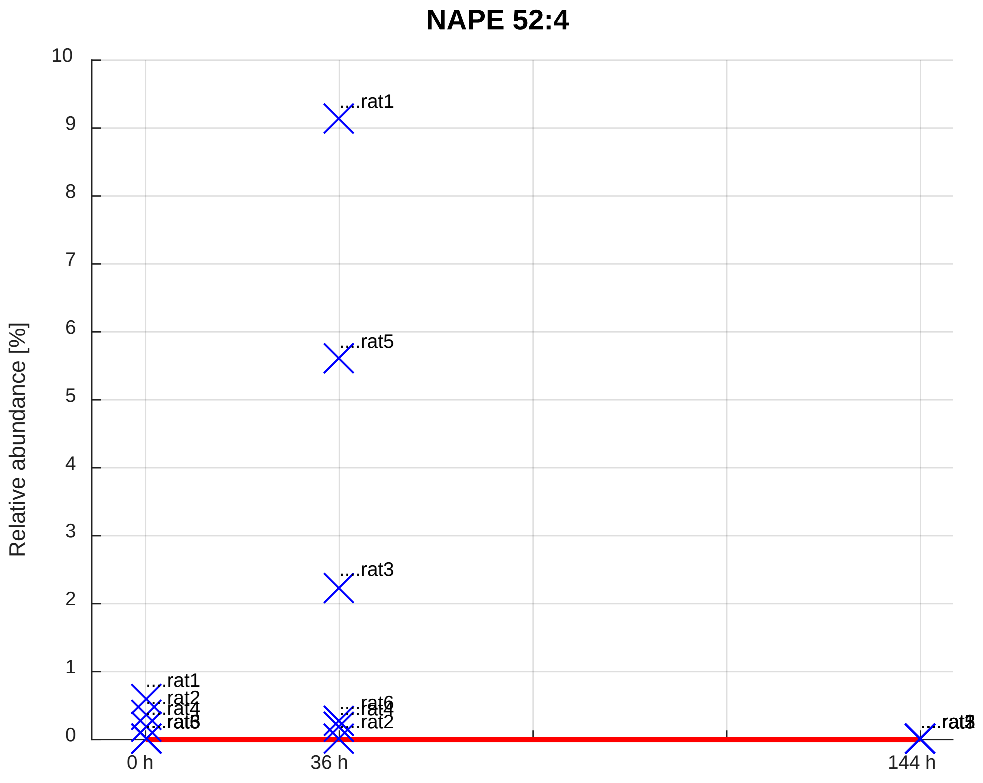

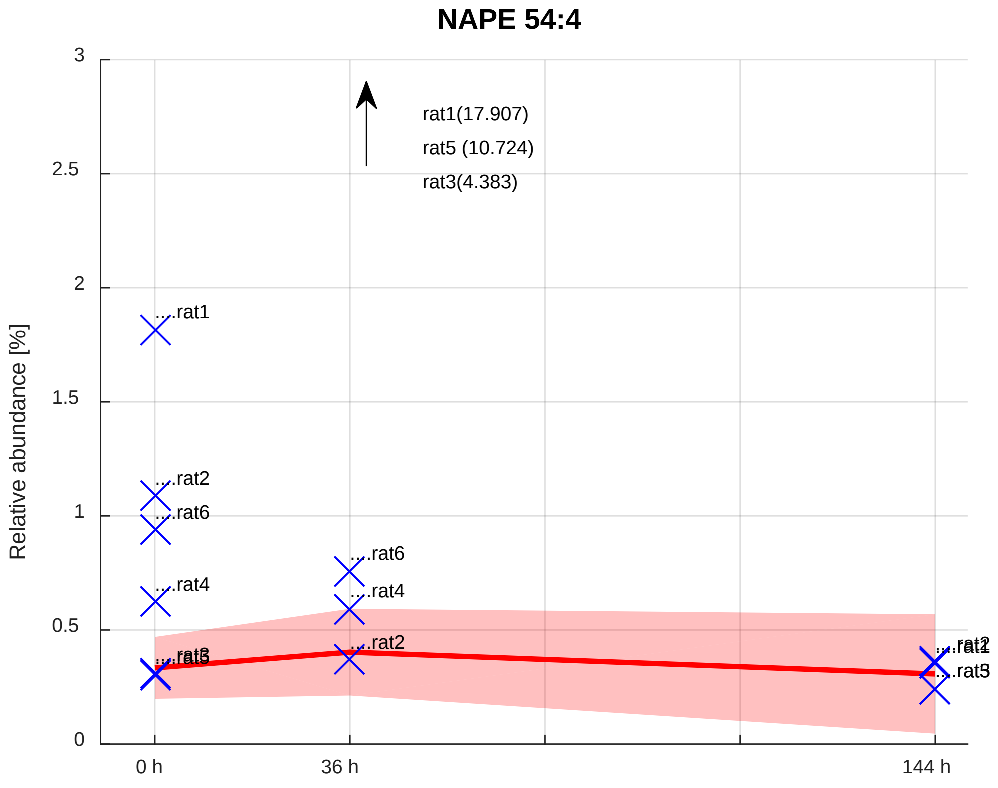

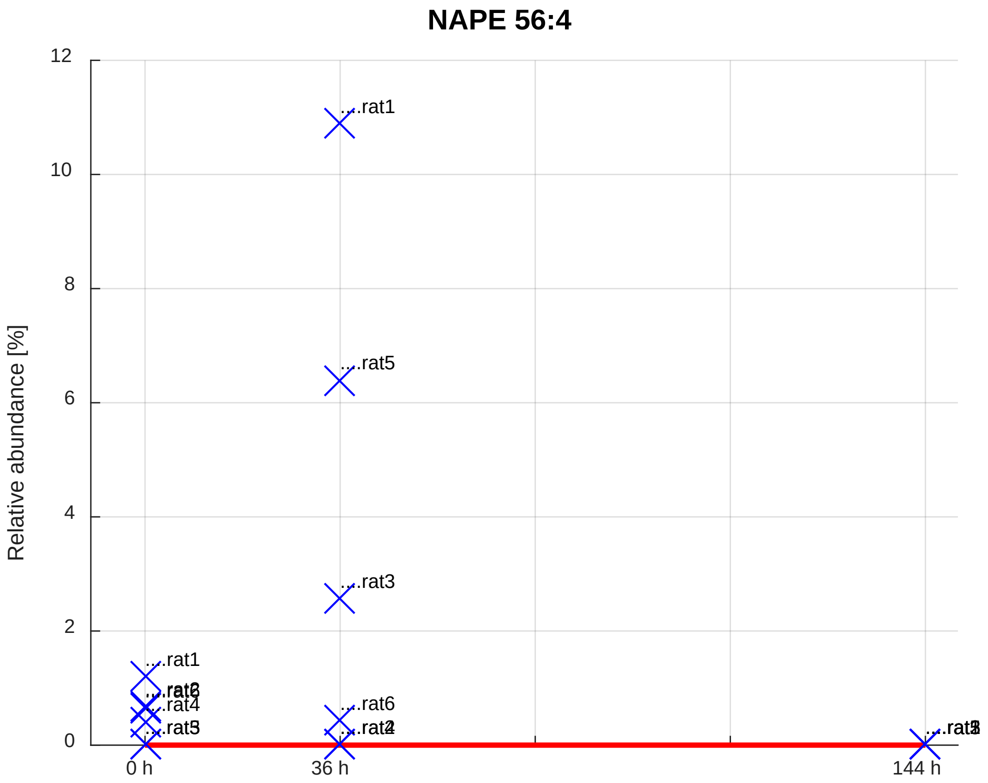
NAPE 56:4
Relative abundance [%]
0
2
4
6
8
10
12
0 h
36 h
144 h
...rat1
...rat5
...rat3
...rat6
...rat4

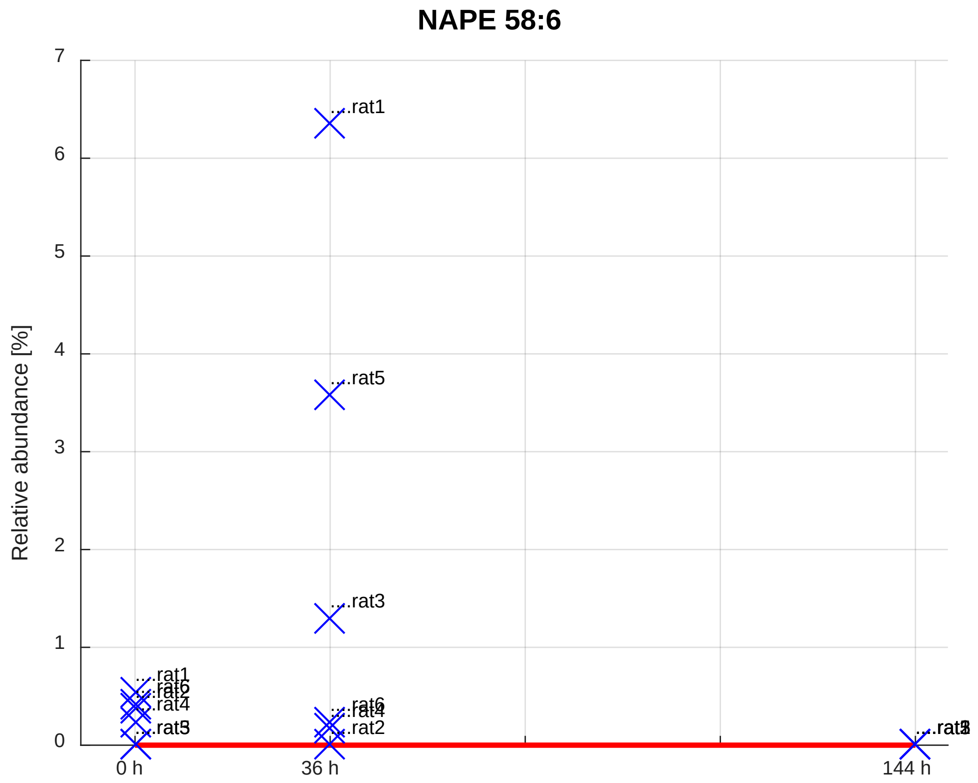
NAPE 58:6
Relative abundance [%]
0
1
2
3
4
5
6
7
0 h
36 h
144 h
...rat1
...rat5
...rat3
...rat4
...rat2

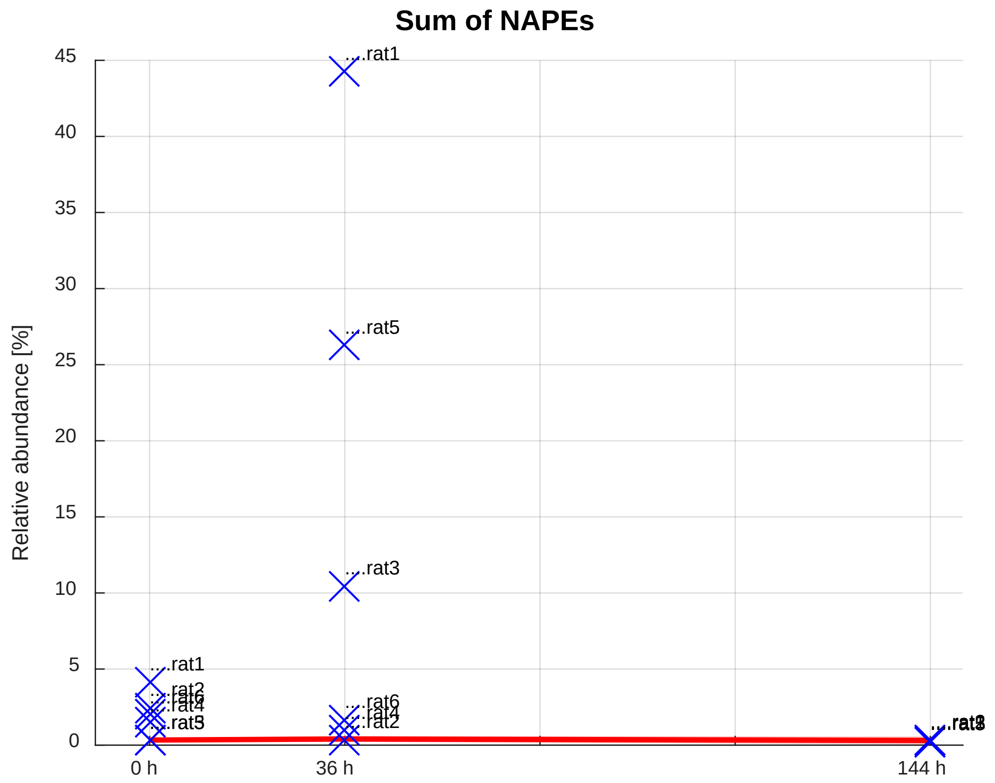
Sum of NAPEs
Relative abundance [%]
45
40
35
30
25
20
15
10
5
0
0 h
36 h
144 h
...rat1
...rat5
...rat3

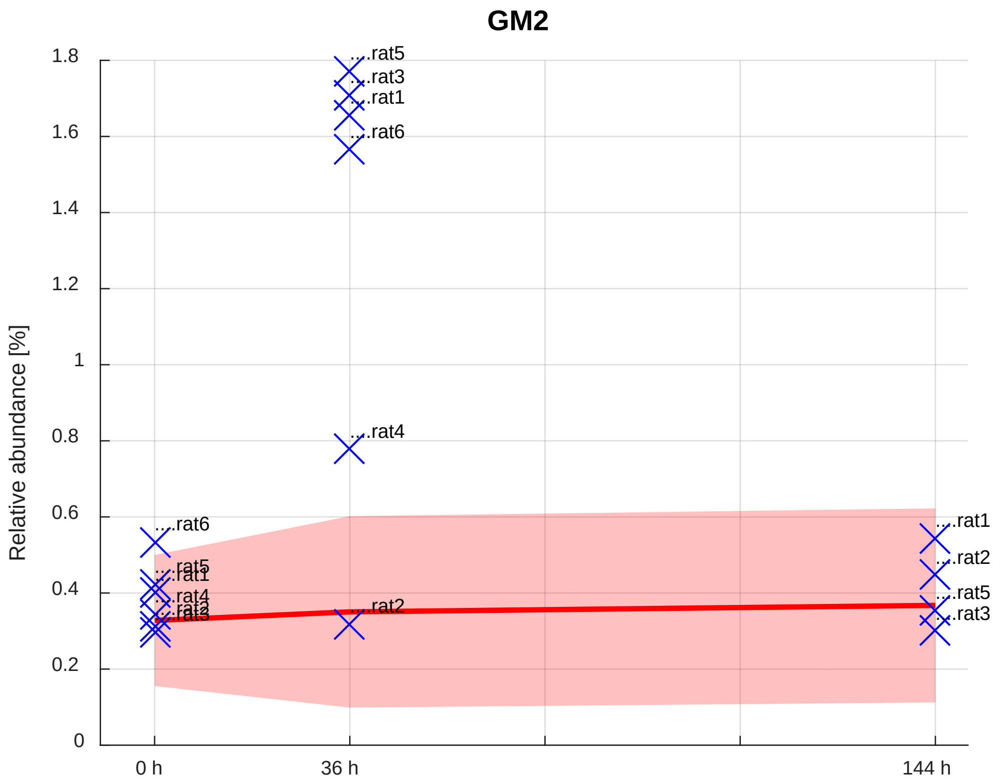
GM2
Relative abundance [%]
1.8
1.6
1.4
1.2
1
0.8
0.6
0.4
0.2
0
0 h
36 h
144 h
...rat5
...rat3
...rat1
...rat6
...rat4
...rat6
...rat2
...rat1
...rat2
...rat5
...rat3

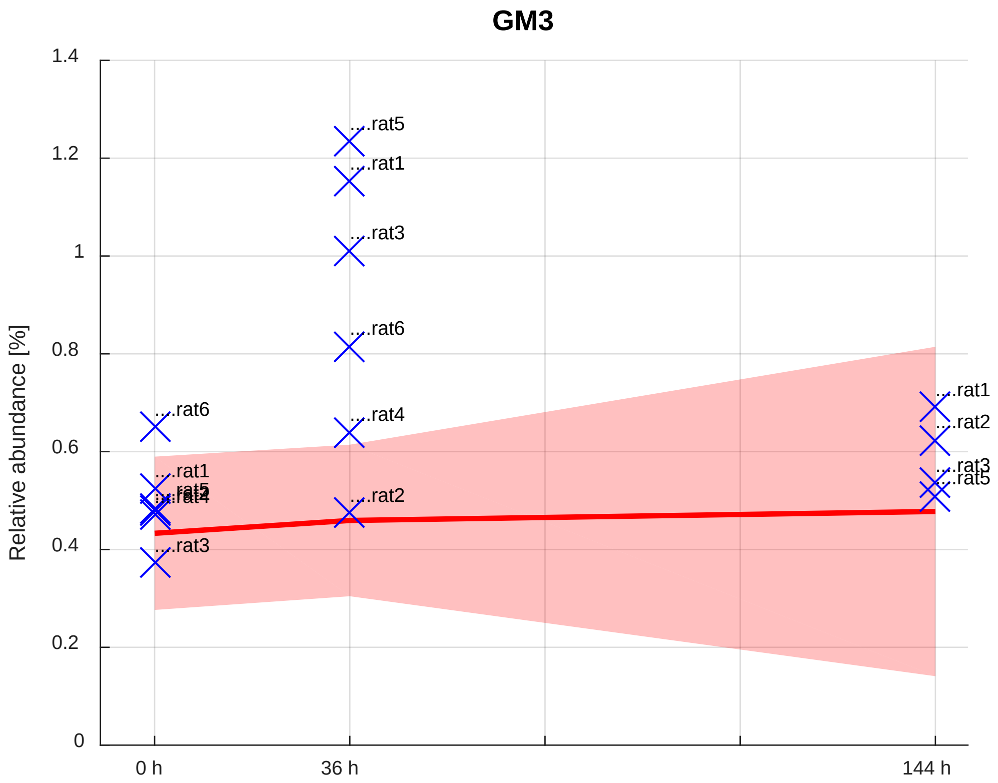

GM3
Relative abundance [%]
1.4
1.2
1
0.8
0.6
0.4
0.2
0
0 h
36 h
144 h
...rat5
...rat1
...rat3
...rat6
...rat4
...rat2
...rat6
...rat1
...rat3
...rat1
...rat2
...rat3
...rat5

Table S1: Lesion size related to cytotoxic ooedema expressed as hemisphere (ipsilateral/contralateral) volume in seven experimental rats. SD and SEM stand for Standard Deviation and Standard Error of the Mean, respectively.

Lesion size/ hemisphere volume (%)		
**rat No.**	**ipsi**	**contra**
**rat3**	7.1	0.0
**rat5**	8.0	0.0
**rat7**	13.8	0.0
**rat9**	13.7	0.0
**rat10**	13.9	0.0
**rat16**	15.7	4.5
**rat18**	11.3	7.5
**mean (%)**	11.9	1.7
**SD (%)**	3.3	3.1
**SEM (%)**	1.2	1.2

Table S2: Characterization of the observed Na^+ and K^+ adducts of phosphocholines (PCs).

Theoretical *m/z*	Measured m/z	Error (ppm)	Formula	Phosphocholine	Adduct
728.5201	728.5197	-0.6	$C_{38}H_{76}NO_8P$	PC (14:0/16:0)	Na^+
744.4940	744.4937	-0.4			K^+
754.5357	754.5352	-0.7	$C_{40}H_{78}NO_8P$	PC (16:0/16:1)	Na^+
770.5097	770.5102	0.7			K^+
756.5514	756.5505	-1.2	$C_{40}H_{80}NO_8P$	PC (16:0/16:0)	Na^+
772.5253	772.5249	-0.6			K^+
782.5670	782.5673	0.4	$C_{42}H_{82}NO_8P$	PC (16:0/18:1)	Na^+
798.5410	798.5411	0.2			K^+
784.5827	784.5793	4.31	$C_{42}H_{84}NO_8P$	PC (16:0/18:0)	Na^+
800.5566	800.5512	5.26			K^+

Table S3: *N*-acylphosphatidylethanolamines (NAPEs) detected in the HI-affected brains at 36 h after the HI insult.

Theoretical *m/z*	Measured *m/z*	Error (ppm)	Formula	Lipid compound	Relative intensity (%)
974.7219	974.7201	1.90	$C_{57}H_{102}NO_9P$	NAPE (52:5)	10
976.7376	976.7356	2.07	$C_{57}H_{104}NO_9P$	NAPE (52:4)	51
998.7219	998.7210	0.93	$C_{59}H_{102}NO_9P$	NAPE (54:7)	7
1000.7376	1000.7366	0.94	$C_{59}H_{104}NO_9P$	NAPE (54:6)	49
1002.7532	1002.7496	3.68	$C_{59}H_{106}NO_9P$	NAPE (54:5)	52
1004.7689	1004.7282	0.68	$C_{59}H_{108}NO_9P$	NAPE (54:4)	100
1006.7845	1006.7875	3.00	$C_{59}H_{110}NO_9P$	NAPE (54:3)	22
1024.7376	1024.7375	0.11	$C_{61}H_{104}NO_9P$	NAPE (56:8)	6
1026.7532	1026.7493	3.86	$C_{61}H_{106}NO_9P$	NAPE (56:7)	25
1030.7845	1030.7822	2.30	$C_{61}H_{110}NO_9P$	NAPE (56:5)	46
1032.8002	1032.7994	0.81	$C_{61}H_{112}NO_9P$	NAPE (56:4)	53
1034.8158	1034.8155	0.35	$C_{61}H_{114}NO_9P$	NAPE (56:3)	11
1052.7689	1052.7663	2.46	$C_{63}H_{108}NO_9P$	NAPE (58:8)	7
1054.7845	1054.7826	1.88	$C_{63}H_{110}NO_9P$	NAPE (58:7)	18
1056.8002	1056.7990	1.09	$C_{63}H_{112}NO_9P$	NAPE (58:6)	37
1060.8315	1060.8323	0.82	$C_{63}H_{116}NO_9P$	NAPE (58:4)	9

Table S4: Statistical analysis of differences between the HII group and control group performed using nonparametric two-tailed Mann–Whitney U test. *P* values indicating statistical significance are in red color.

	NAPE 52:4	NAPE 54:4	NAPE 56:4	NAPE 58:6	Sum of NAPEs	GM2	GM3
Time 0 h	0.182	0.180	0.061	0.061	0.180	0.132	0.240
Time 36 h	**0.015**	**0.026**	0.061	**0.015**	**0.026**	**0.026**	**0.004**
Time 144 h	1.000	0.486	1.000	1.000	0.486	0.486	0.200

Table S5: Statistical analysis of differences between the HII group and control group performed using parametric two-tailed t-tests. *P* values indicating statistical significance are in red color.

	NAPE 52:4	NAPE 54:4	NAPE 56:4	NAPE 58:6	Sum of NAPEs	GM2	GM3
Time 0h	0.115	0.080	**0.048**	**0.036**	0.059	0.220	0.177
Time 36h	0.113	0.124	0.120	0.121	0.120	**0.012**	**0.016**
Time 144h	NaN	0.882	NaN	NaN	0.882	0.528	0.125

Table S6: Tissue reduction at 144 hours after the HI insult recorded by MRI.

HII		SHAM	
**rat No.**	**144h**	**rat No.**	**144h**
**rat3**	8.9%	**rat12S**	0.7%
**rat5**	11.2%	**rat13S**	2.7%
**rat7**	15.6%	**rat14S**	2.4%
**rat9**	18.1%	**rat15S**	0.1%
**rat10**	9.3%	**rat17S**	3.3%
**rat16**	17.1%	**rat19S**	4.1%
**rat18**	11.3%		
**mean (%)**	13.1	**mean (%)**	2.2
**SD (%)**	3.8	**SD (%)**	1.5
**SEM (%)**	1.4	**SEM (%)**	0.6

Table S7: Multiple parametric testing of the time effect based on one-way ANOVA. *P* values indicating statistical significance are in red color.

	**NAPE 52:4**	**NAPE 54:4**	**NAPE 56:4**	**NAPE 58:6**	**Sum of NAPEs**	**GM2**	**GM3**
Time 0h ver. 36h	0.188	0.228	0.276	0.269	0.235	**0.003**	**0.013**
Time 0h ver. 144h	1.000	1.000	1.000	1.000	1.000	1.000	1.000
Time 36h ver.144h	0.219	0.233	0.240	0.241	0.233	**0.010**	0.107

Table S8: Multiple nonparametric testing of the time effect based on Kruskal-Wallis method. *P* values indicating statistical significance are in red color.

	**NAPE 52:4**	**NAPE 54:4**	**NAPE 56:4**	**NAPE 58:6**	**Sum of NAPEs**	**GM2**	**GM3**
Time 0h ver. 36h	0.521	0.825	1.000	1.000	0.825	**0.046**	**0.039**
Time 0h ver. 144h	0.639	0.278	0.353	0.268	0.278	1.000	0.667
Time 36h ver.144h	0.041	**0.024**	0.096	0.052	**0.024**	0.248	0.947

Table S9: Technical variability expressed as Coefficients of Variation (CV) for each group of technical replications.

**HII group**	**NAPE 52:4**	**NAPE 54:4**	**NAPE 56:4**	**NAPE 58:6**	**Sum of NAPEs**	**GM2**	**GM3**
Time 0h	0.018±0.010	0.035±0.007	0.033±0.013	0.042±0.021	0.043±0.008	0.094±0.012	0.114±0.014
Time 36h	0.077±0.031	0.068±0.012	0.034±0.012	0.057±0.017	0.072±0.015	0.097±0.026	0.078±0.014

Time 144h	0	0.034±0.007	0	0	0.034±0.007	0.068±0.017	0.098±0.025

**Control group**	**NAPE 52:4**	**NAPE 54:4**	**NAPE 56:4**	**NAPE 58:6**	**Sum of NAPEs**	**GM2**	**GM3**
Time 0h	0	0.043±0.007	0	0	0.043±0.007	0.093±0.012	0.135±0.020
Time 36h	0	0.048±0.013	0	0	0.048±0.013	0.082±0.020	0.121±0.040
Time 144h	0	0.043±0.009	0	0	0.043±0.009	0.053±0.013	0.055±0.024

Table S10: Biological variability expressed as Coefficient of Variation (CV).

**HII group**	**NAPE 52:4**	**NAPE 54:4**	**NAPE 56:4**	**NAPE 58:6**	**Sum of NAPEs**	**GM2**	**GM3**
Time 0h	1.286	0.677	0.942	0.859	0.817	0.228	0.182
Time 36h	1.278	1.233	1.309	1.313	1.272	0.465	0.335
Time 144h	0.000	0.218	0.000	0.000	0.218	0.262	0.140
**Control group**	**NAPE 52:4**	**NAPE 54:4**	**NAPE 56:4**	**NAPE 58:6**	**Sum of NAPEs**	**GM2**	**GM3**
Time 0h	0.000	0.146	0.000	0.000	0.146	0.189	0.130
Time 36h	0.000	0.170	0.000	0.000	0.170	0.259	0.121
Time 144h	0.000	0.239	0.000	0.000	0.239	0.195	0.198

Table S11: Percentage of the biological variability from the total variability for each dataset.

**HII group**	**NAPE 52:4**	**NAPE 54:4**	**NAPE 56:4**	**NAPE 58:6**	**Sum of NAPEs**	**GM2**	**GM3**
Time 0h	99.78%	99.85%	99.74%	99.00%	99.77%	86.23%	75.67%
Time 36h	99.50%	99.62%	99.68%	99.66%	99.62%	95.28%	93.20%
Time 144h		97.09%			97.09%	94.72%	67.26%
**Control group**	**NAPE 52:4**	**NAPE 54:4**	**NAPE 56:4**	**NAPE 58:6**	**Sum of NAPEs**	**GM2**	**GM3**
Time 0h		91.72%			91.72%	80.91%	52.66%
Time 36h		90.22%			90.22%	88.41%	39.05%
Time 144h		96.23%			96.23%	93.83%	92.71%

Table S12: *P*-values of the Lilliefors corrected Kolmogorov-Smirnov test of the normality. Values indicating statistical significance (if the *P*-value rejected normal distribution) are in red.

**HII group**	**NAPE 52:4**	**NAPE 54:4**	**NAPE 56:4**	**NAPE 58:6**	**Sum of NAPEs**	**GM2**	**GM3**
Time 0h	0.148	0.500	0.500	0.500	0.500	0.500	0.450
Time 36h	0.243	0.246	0.304	0.210	0.267	**0.034**	0.500
Time 144h	**<0.001**	0.202	**<0.001**	**<0.001**	0.202	0.500	0.500
**Control group**	**NAPE 52:4**	**NAPE 54:4**	**NAPE 56:4**	**NAPE 58:6**	**Sum of NAPEs**	**GM2**	**GM3**
Time 0h	**<0.001**	0.182	**<0.001**	**<0.001**	0.182	0.026	0.095
Time 36h	**<0.001**	0.500	**<0.001**	**<0.001**	0.500	0.070	0.126
Time 144h	**<0.001**	0.194	**<0.001**	**<0.001**	0.194	0.386	0.500

Table S13: Values (mean ± SEM) of the relative abundance of the NAPE representatives, GM2 and GM3 of the 0, 36 and 144 time intervals.

**HII group**	**NAPE 52:4**	**NAPE 54:4**	**NAPE 56:4**	**NAPE 58:6**	**Sum of NAPEs**	**GM2**	**GM3**
Time 0h	0.194±0.096	0.847±0.220	0.483±0.175	0.261±0.086	1.784±0.560	0.386±0.036	0.497±0.040
Time 36h	2.910±1.430	5.789±2.743	3.375±1.697	1.936±0.977	14.010±6.845	1.300±0.237	0.888±0.118
Time 144h	0	0.300±0.030	0	0	0.300±0.030	0.411±0.050	0.590±0.046
**Control group**	**NAPE 52:4**	**NAPE 54:4**	**NAPE 56:4**	**NAPE 58:6**	**Sum of NAPEs**	**GM2**	**GM3**
Time 0h	0	0.334±0.020	0	0	0.334±0.020	0.328±0.026	0.433±0.030
Time 36h	0	0.403±0.028	0	0	0.403±0.028	0.350±0.037	0.459±0.034
Time 144h	0	0.307±0.034	0	0	0.307±0.034	0.367±0.033	0.478±0.044
